# Genomic insights into neonicotinoid sensitivity in the solitary bee *Osmia bicornis*

**DOI:** 10.1371/journal.pgen.1007903

**Published:** 2019-02-04

**Authors:** Katherine Beadle, Kumar Saurabh Singh, Bartlomiej J. Troczka, Emma Randall, Marion Zaworra, Christoph T. Zimmer, Angela Hayward, Rebecca Reid, Laura Kor, Maxie Kohler, Benjamin Buer, David R. Nelson, Martin S. Williamson, T. G. Emyr Davies, Linda M. Field, Ralf Nauen, Chris Bass

**Affiliations:** 1 College of Life and Environmental Sciences, Biosciences, University of Exeter, Penryn Campus, Penryn, Cornwall, United Kingdom; 2 Department of Biointeractions and Crop Protection, Rothamsted Research, Harpenden, United Kingdom; 3 Bayer AG, Crop Science Division, R&D, Monheim, Germany; 4 Department of Microbiology, Immunology and Biochemistry, University of Tennessee Health Science Center, Memphis, TN, United States of America; National Institute of Genetics, JAPAN

## Abstract

The impact of pesticides on the health of bee pollinators is determined in part by the capacity of bee detoxification systems to convert these compounds to less toxic forms. For example, recent work has shown that cytochrome P450s of the CYP9Q subfamily are critically important in defining the sensitivity of honey bees and bumblebees to pesticides, including neonicotinoid insecticides. However, it is currently unclear if solitary bees have functional equivalents of these enzymes with potentially serious implications in relation to their capacity to metabolise certain insecticides. To address this question, we sequenced the genome of the red mason bee, *Osmia bicornis*, the most abundant and economically important solitary bee species in Central Europe. We show that *O*. *bicornis* lacks the CYP9Q subfamily of P450s but, despite this, exhibits low acute toxicity to the *N*-cyanoamidine neonicotinoid thiacloprid. Functional studies revealed that variation in the sensitivity of *O*. *bicornis* to *N*-cyanoamidine and *N*-nitroguanidine neonicotinoids does not reside in differences in their affinity for the nicotinic acetylcholine receptor or speed of cuticular penetration. Rather, a P450 within the CYP9BU subfamily, with recent shared ancestry to the Apidae CYP9Q subfamily, metabolises thiacloprid *in vitro* and confers tolerance *in vivo*. Our data reveal conserved detoxification pathways in model solitary and eusocial bees despite key differences in the evolution of specific pesticide-metabolising enzymes in the two species groups. The discovery that P450 enzymes of solitary bees can act as metabolic defence systems against certain pesticides can be leveraged to avoid negative pesticide impacts on these important pollinators.

## Author summary

Bees have evolved sophisticated metabolic systems to detoxify the natural toxins encountered in their environment. Recent work has shown that specific enzymes (cytochrome P450s) in these biotransformation pathways can be recruited to protect honey bees and bumblebees against certain synthetic insecticides, including some neonicotinoids. However, it is unclear if solitary bees that carry out important pollination services have equivalent enzymes that play a key role in defining their sensitivity to insecticides. In this study we show that the genome of the solitary bee, *Osmia bicornis*, lacks the subfamily of cytochrome P450 enzymes that break down certain neonicotinoids in eusocial bees. Despite this, *O*. *bicornis* exhibits marked tolerance to the neonicotinoid thiacloprid as a result of efficient metabolism by a P450 enzyme from an alternative subfamily. The discovery that *O*. *bicornis* has key detoxification enzymes that determine its sensitivity to neonicotinoids can be leveraged to safeguard the health of this important pollinator.

## Introduction

Bee pollinators encounter a wide range of natural and synthetic xenobiotics while foraging or in the hive, including phytochemicals, mycotoxins produced by fungi, and pesticides [1]. Understanding the toxicological outcomes of bee exposure to these chemicals, in isolation or combination, is essential to safeguard bee health and the ecosystem services they provide. Like other insects, bees have sophisticated metabolic systems that mediate the conversion of harmful xenobiotics to less toxic forms, and these detoxification pathways can be critically important in defining their sensitivity to xenobiotics including pesticides [2]. In an important recent example of this cytochrome P450 enzymes belonging to the CYP9Q subfamily were shown to play a key role in determining the sensitivity of honey bees and bumblebees to neonicotinoid insecticides [3]. Prior work on honey bees showed that the same P450s also provide protection against the toxic effects of certain insecticides from the pyrethroid and organophosphate classes that are used for the control of parasitic *Varroa* mites [4]. Taken together these studies suggest CYP9Q P450s may be important generalist detoxification enzymes. To date our understanding of bee biochemical defence systems stems from work on eusocial species, namely honey bees and bumblebees, with much less attention given to solitary species. However, the majority of bee species are solitary, and there is increasing awareness of the importance of solitary bees as pollinators of wild plants and certain crops [5–8]. It is currently unknown to what extent the discoveries on the metabolic systems of honey bees and bumblebees extend to solitary bees, and thus if the use of eusocial species as a proxy for solitary species in ecotoxicological studies is reliable.

The red mason bee, *Osmia bicornis* (syn. *O*. *rufa*) (Hymenoptera: Megachilidae) is the most abundant and economically important solitary bee species in Central Europe [9]. This species pollinates a range of wild plants and is also used for commercial pollination, particularly of fruit crops (almond, peach, apricot, plum, cherry, apple and pear). Understanding *O*. *bicornis*-pesticide interactions is particularly important as it has been recommended as a solitary bee model for the registration of pesticides in Europe [10]. However, to date, investigations on this topic have been hampered by a lack of genomic and transcriptomic resources for this species.

In this study we addressed this knowledge and resource gap by generating a high quality genome assembly of *O*. *bicornis*. We then exploited this genomic resource to compare the complement of P450 genes in *O*. *bicornis* with that of other bee species, and identify P450 enzymes that are important determinants of *O*. *bicornis* sensitivity to neonicotinoid insecticides.

## Results

### The genome of *O*. *bicornis* lacks the CYP9Q subfamily of P450s observed in eusocial bees

To generate a high-quality genome assembly of *O*. *bicornis* we sequenced genomic DNA extracted from a single haploid male bee using a combination of Illumina paired-end and mate-pair libraries. Additional RNA sequencing (RNAseq) of male and female bees was also performed in order to improve the quality of subsequent gene prediction. DNAseq data was assembled to generate an *O*. *bicornis* genome of 212.9 Mb consistent with genome size estimates derived from k-mer analysis of the raw reads (S1 Table). The final assembly comprised 10,223 scaffolds > 1 kb with a scaffold and contig N50 of 604 kb and 303 kb respectively (S2 Table). Structural genome annotation using a workflow incorporating RNAseq data predicted a total of 14,858 protein-coding genes encoding 18,479 total proteins (S3 Table). The completeness of the gene space in the assembled genome was assessed using the Benchmarking Universal Single-Copy Orthologues (BUSCO) pipeline [11] with greater than 99% of Arthropoda and Insecta test genes identified as complete in the assembly (S4 Table). Approximately 78% of the predicted genes could be assigned functional annotation based on BLAST searches against the non-redundant protein database of NCBI (S1 Fig).

**Fig 1:**
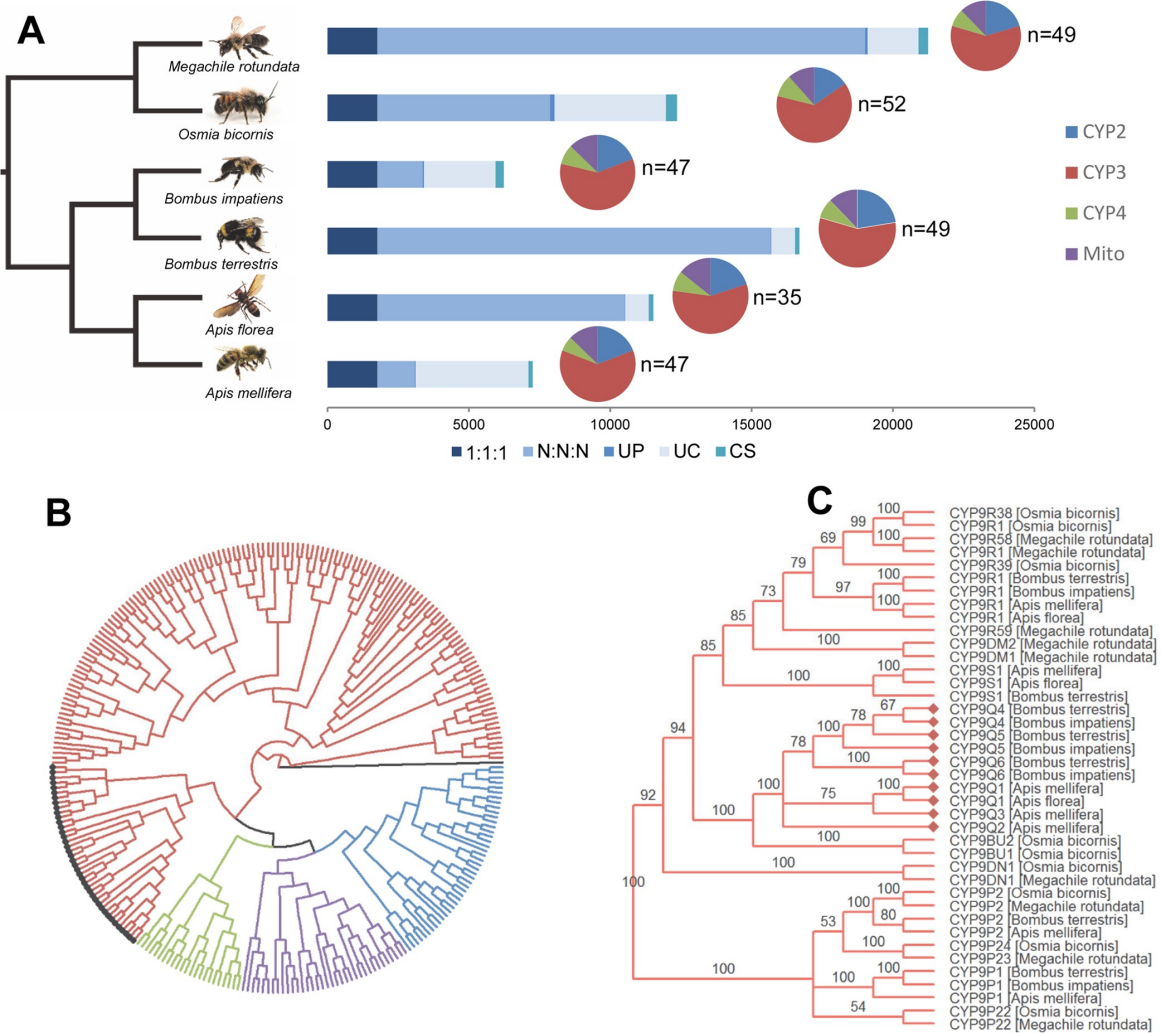
Comparison of the CYPome of *O*. *bicornis* with other bee species. (**A**) Ortholog analysis of *O*. *bicornis* with five other bee species. 1:1:1 indicates common orthologs with the same number of copies in different species, N:N:N indicates common orthologs with different copy numbers in different species, UP indicates species specific paralogs, UC indicates all genes which were not assigned to a gene family, CS indicates clade specific genes. Pie charts show the percentage of genes in the CYPome of each bee species in the CYP2, 3, 4 and mitochondrial clade. (**B**) Rooted maximum likelihood consensus phylogenetic tree of the CYPome of the same species shown in panel A. Genes are coloured according to their adscription to different P450 clades. (**C**) Maximum likelihood phylogenetic tree of the CYP9 family of P450s in the same species, P450s belonging to the CYP9Q subfamily are highlighted using filled diamonds.

**Fig 2:**
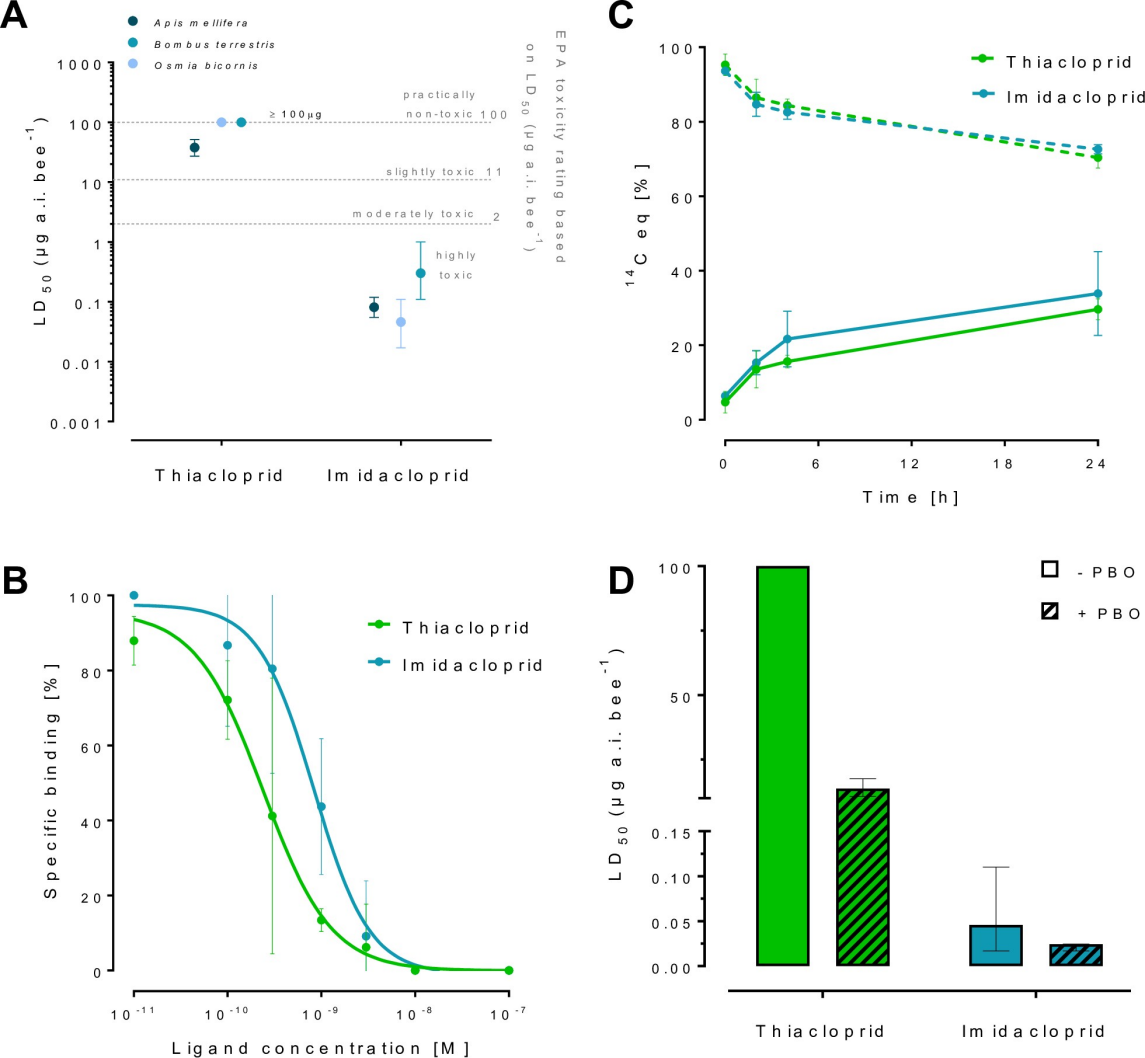
Toxicodynamics and pharmacokinetics of neonicotinoid sensitivity in *O*. *bicornis*. (**A**) LD_50_ values for imidacloprid and thiacloprid in insecticide bioassays for *O*. *bicornis*, for comparison data is also shown for *A*. *mellifera* and *B*. *terrestris*. Sensitivity thresholds are depicted according to EPA toxicity ratings [45]. Data for *A*. *mellifera* is taken from [13,14], data for *B*. *terrestris* is taken from [3]. Error bars display 95% CLs (n = 4). (**B**) Specific binding of thiacloprid and imidacloprid to *O*. *bicornis* nAChRs. Error bars display standard deviation (n = 3). (**C**) Penetration of radiolabelled thiacloprid and imidacloprid through the cuticle of *O*. *bicornis*. The percentage of the initial ^14^C-imidacloprid and ^14^C-thiacloprid dose recovered by external cuticular rinsing over 24 hours is shown by dashed lines. The percentage of ^14^C-imidacloprid and ^14^C-thiacloprid recovered from combusted bees (i.e. internalized compound) is shown by solid lines. Error bars display standard deviation (n = 3). (**D**) Sensitivity of *O*. *bicornis* to imidacloprid and thiacloprid before and after pre-treatment with the insecticide synergist PBO (piperonyl butoxide). Error bars display 95% CLs (n = 3).

The gene repertoire of *O*. *bicornis* was compared with other colony forming (*Apis mellifera*, *Apis florea*, *Bombus terrestris* and *Bombus impatiens*) and solitary bee species (*Megachile rotundata*) by orthology inference (Fig 1A). The combined gene count of these species was 101,561 of which ~90% were assigned to 11,184 gene families. Of these 8,134 gene families were present in *O*. *bicornis* and all other species, and a total of 163 gene families were specific to *O*. *bicornis* compared to 21–97 in the other bee species (Fig 1A). Genes encoding cytochrome P450s were identified from orthogroups, and individual bee genomes (see methods), and the complete complement of P450s in each bee genome (the CYPome) was curated and named by the P450 nomenclature committee (S5 Table). The genome of *O*. *bicornis* contains 52 functional P450s (Fig 2B and S2 Fig), a gene count consistent with the other bee species and reduced in comparison to other insects, even including other hymenoptera [2]. As for other insect species bee P450 genes group into four main clades (CYP2, CYP3, CYP4 and mitochondrial clans) of which by far the largest (comprising 33 P450s in *O*. *bicornis*) is the CYP3 clan of CYP6, CYP9 and CYP336 (Fig 1A and 1B, S2 Fig). Phylogenetic comparison of the CYP9 family within this clade in *O*. *bicornis* and 11 other bee species [12] revealed that *O*. *bicornis* lacks the CYP9Q subfamily found in eusocial bee species that has been shown to define the sensitivity of honey bees and bumblebees to neonicotinoids (Fig 1C, S3 Fig) [3]. The most closely related subfamily in *O*. *bicornis* was CYP9BU (represented by *CYP9BU1* and *CYP9BU2*), a newly described subfamily, that appears to share a relatively recent common ancestor with the CYP9Q subfamily (Fig 1C, S3 Fig).

### Despite the lack of CYP9Q P450s *O*. *bicornis* exhibits marked variation in sensitivity to *N*-nitroguanidine and *N*-cyanoamidine neonicotinoids

In the absence of the CYP9Q subfamily of P450s it might be expected that *O*. *bicornis* would be more sensitive to neonicotinoids (especially *N*-cyanoamidine compounds) than honey bees and bumblebees. To test this we performed acute contact insecticide bioassays using imidacloprid and thiacloprid as representatives of *N*-nitroguanidine and *N*-cyanoamidine neonicotinoids respectively. Significant differences were found in the tolerance of *O*. *bicornis* to the two compounds with adult female bees >2,000-fold more sensitive to imidacloprid (LD_50_ of 0.046 μg/bee) than thiacloprid (LD_50_ of >100 μg/bee) (Fig 2A). These values are similar to those reported for honey bees and bumblebees [3,13,14] with imidacloprid classified as ‘highly toxic’ to *O*. *bicornis* according to the categories of the U.S. Environmental Protection Agency, but thiacloprid classified as ‘practically non-toxic’ upon contact exposure (Fig 2A). Thus these results clearly show that, despite the lack of CYP9Q P450s, *O*. *bicornis* has high levels of tolerance to the *N*-cyanoamidine neonicotinoid thiacloprid.

### Variation in the sensitivity of *O*. *bicornis* to thiacloprid and imidacloprid does not reside in differences in their affinity for the receptor or speed of cuticular penetration

The molecular basis of the profound variation in the sensitivity of *O*. *bicornis* to imidacloprid and thiacloprid could reside in differences in: a) their affinity for the target-site, the nicotinic acetylcholine receptor (nAChR), b) their speed of penetration through the cuticle, or c) the efficiency of their metabolism. We first examined the affinity of the two compounds for the target-site using radioligand binding assays performed on *O*. *bicornis* head membrane preparations, and examined the displacement of tritiated imidacloprid by both unlabelled imidacloprid and thiacloprid. Both compounds bound with nM affinity—IC_50_ of 8.3 nM [95% Cl 4.6, 15.1] for imidacloprid and 2.4 nM [95% Cl, 1.4, 4.1] for thiacloprid (Fig 2B). These values suggest that thiacloprid binds with higher affinity than imidacloprid, however, no significant difference was observed between the slopes of the regression lines of the two compounds (p = 0.3). This finding clearly demonstrates that the tolerance of *O*. *bicornis* to thiacloprid relative to imidacloprid is not a consequence of a reduced affinity of the former for the nAChR.

To explore the rate of penetration of these two compounds through the cuticle of *O*. *bicornis* the uptake of [^14^C]imidacloprid and [^14^C]thiacloprid after application to the dorsal thorax was compared. No significant differences were observed in the amount of radiolabelled thiacloprid and imidacloprid recovered from the cuticle or acetone combusted whole bees at any time point post-application (the final uptake through the cuticle after 24h was 27% of [^14^C]imidacloprid and 28% of [^14^C]thiacloprid, Fig 2C). Thus, the differential sensitivity of *O*. *bicornis* to imidacloprid and thiacloprid is not a result of variation in their speed of penetration through the cuticle.

### The tolerance of *O*. *bicornis* to thiacloprid is mediated by CYP9BU P450s

Insecticide synergists that inhibit detoxification enzymes have been used to explore the role of metabolism in the tolerance of honey bees and bumblebees to certain neonicotinoids. Specifically, the use of the P450 inhibitor piperonyl butoxide (PBO) provided strong initial evidence that P450s underpin the tolerance of both bee species to *N*-cyanoamidine neonicotinoids [3,15]. We therefore examined the effect of PBO pre-treatment on the sensitivity of *O*. *bicornis* to thiacloprid and imidacloprid in insecticide bioassays. No significant difference was observed in the sensitivity of *O*. *bicornis* to imidacloprid with or without PBO, however, bees pre-treated with PBO became >7-fold more sensitive to thiacloprid (Fig 2D), suggesting that P450s play an important role in defining the sensitivity of *O*. *bicornis* to neonicotinoids.

**Fig 3:**
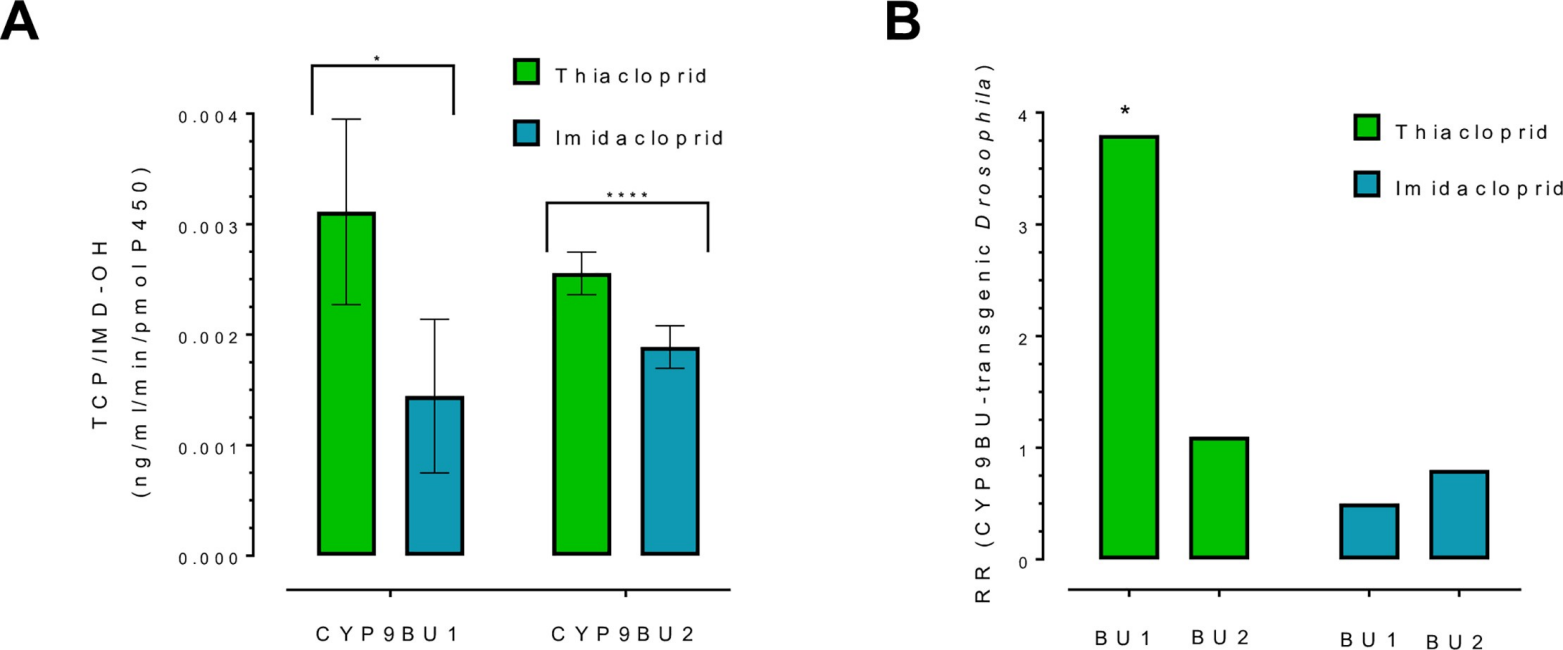
Identification of neonicotinoid metabolising P450s in *O*. *bicornis*. (A) Metabolism of thiacloprid and imidacloprid by recombinantly expressed CYP9BU1 and CYP9BU2. Production of 5-hydroxy thiacloprid and 5-hydroxy imidacloprid is displayed per pmol of P450 (*P<0.05, ****P<0.0001; paired t test). Error bars display standard deviation (n = 3). (B) Sensitivity of transgenic flies expressing CYP9BU1 and CYP9BU2 to thiacloprid and imidacloprid in insecticide bioassays. Data is expressed as resistance ratio (RR) compared to a control line (flies of the same genetic background but without the transgene). Significant changes in sensitivity between control and transgenic lines are indicated by an asterisk and are based on non-overlapping 95% fiducial limits of LC50 values (n = 5). See also S6 Table.

As detailed above, based on phylogeny, CYP9BU1 and CYP9BU2 are clearly the most closely related P450s in *O*. *bicornis* to the Apidae CYP9Q subfamily which metabolise thiacloprid in honey bees and bumblebees (Fig 1C, S3 Fig). We therefore examined the capacity of these P450s to metabolise thiacloprid and imidacloprid *in vitro* by individually coexpressing them with house fly cytochrome P450 reductase (CPR) in an insect cell line. Incubation of microsomal preparations containing each P450 and CPR with either thiacloprid or imidacloprid, and analysis of the metabolites produced by liquid chromatography tandem mass spectrometry (LC-MS/MS), revealed that both CYP9BU1 and CYP9BU2 metabolise these compounds to their hydroxylated forms (5-hydroxy thiacloprid and 5-hydroxy imidacloprid respectively) (Fig 3A). Both P450s metabolised thiacloprid with significantly greater efficiency than imidacloprid (Fig 3A) consistent with the relative sensitivity of *O*. *bicornis* to these compounds. To provide additional evidence that these P450s confer tolerance to *N*-cyanoamidine neonicotinoids *in vivo*, we created transgenic lines of *Drosophila melanogaster* expressing *CYP9BU1*, or *CYP9BU2* and examined their sensitivity to imidacloprid and thiacloprid. Flies expressing the *CYP9BU1* transgene were ~4 times less sensitive to thiacloprid than control flies of the same genetic background without the transgene in insecticide bioassays (Fig 3B, S6 Table). In contrast flies expressing *CYP9BU2* showed no significant resistance to thiacloprid. In bioassays using imidacloprid no significant differences in sensitivity were observed between flies with either of the two transgenes and control flies. These results demonstrate that the transcription of *CYP9BU1* confers intrinsic tolerance to thiacloprid *in vivo*.

### Expression profiling of *O*. *bicornis* P450s reveals constitutive expression of *CYP9BU1* in tissues associated with xenobiotic detoxification

Characterising when and where the neonicotinoid-metabolising P450s identified in this study are expressed is an important step in understanding their capacity to protect *O*. *bicornis in vivo*. To investigate this, we 1) explored changes in their expression in response to exposure to sublethal doses of imidacloprid and thiacloprid, and 2) examined their expression in tissues that are involved in xenobiotic detoxification, or are sites of insecticide action.

**Fig 4:**
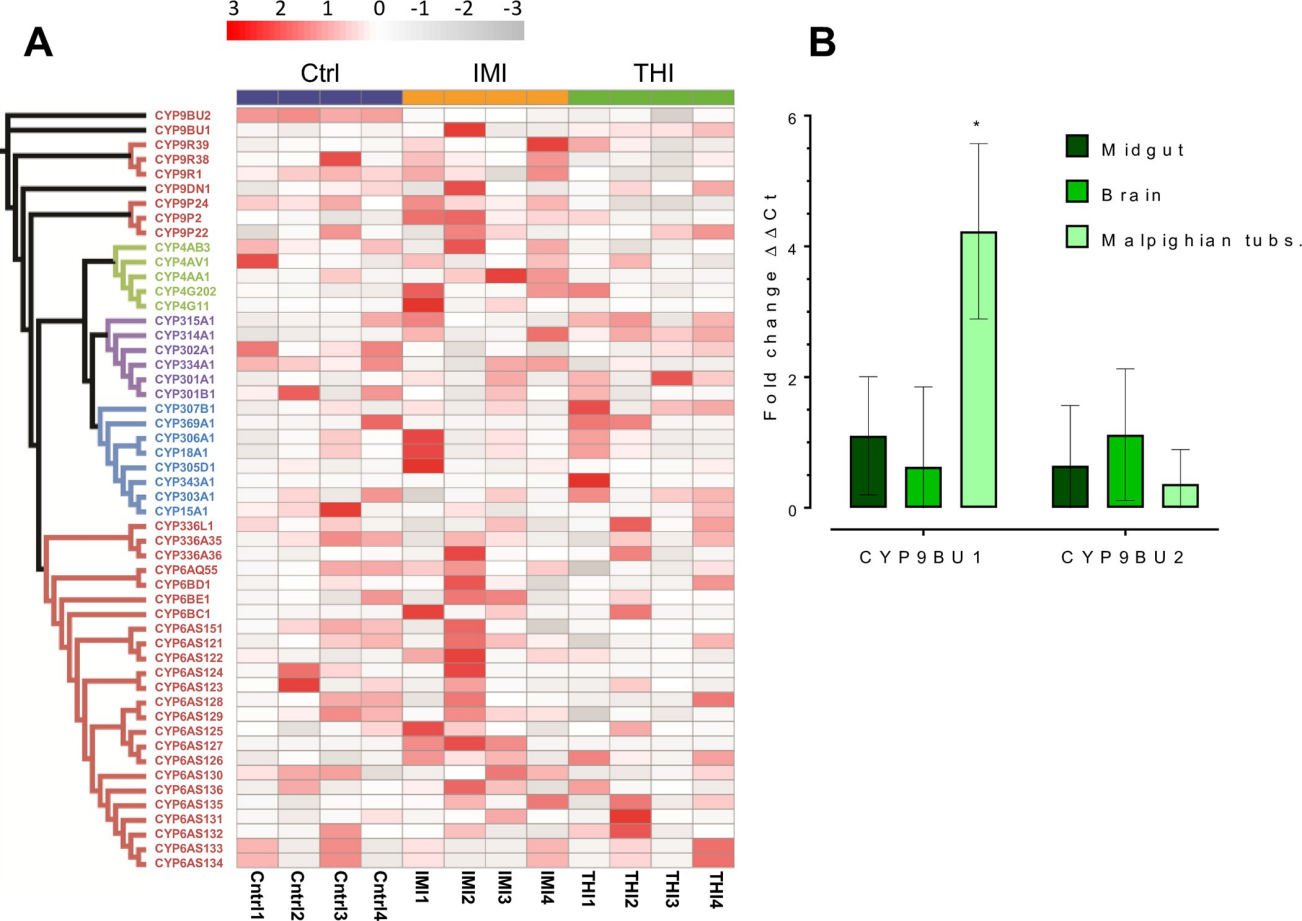
Expression of *O*. *bicornis* P450s after exposure to neonicotinoids and in different tissues. (A) Expression heat map of *O*. *bicornis* P450s after exposure of female bees to imidacloprid (IMI), thiacloprid (THI) or insecticide dilutent (Ctrl). Expression in each of the four replicates per treatment is derived from scaled FPKM values for each P450 transcript. A maximum likelihood tree of *Osmia bicornis* P450s is shown to the left of the heatmap. (B) Relative expression (fold change) of *O*. *bicornis* thiacloprid metabolising CYP9 genes in different tissues of female bees measured by quantitative PCR. Significant differences (p<0.01) in expression between tissues is denoted using an asterisk above bars as determined by one-way ANOVA with post hoc Tukey HSD.

To investigate if the expression of any genes encoding P450s could be induced by neonicotinoid exposure RNAseq was performed on adult female *O*. *bicornis* 24 h after exposure to the LD_10_ of thiacloprid, imidacloprid or the solvent used to dissolve insecticides alone (as a control). Differentially expressed genes (corrected p value of <0.05) between control and treatments were identified and are shown in full in S7 Table and S8 Table. In general, changes in gene expression were modest with just 27 genes significantly upregulated after imidacloprid exposure and 16 genes upregulated after thiacloprid exposure. The function of these differentially expressed genes was either unknown or is unrelated to xenobiotic detoxification, and no P450 showed a significant increase in expression upon exposure to either neonicotinoid (Fig 4A, S7 Table and S8 Table). These findings suggest that constitutive rather than induced expression of the P450s identified in this study is more important in their role in pesticide detoxification.

The expression of neonicotinoid-metabolising P450s in the brain, midgut and Malpighian tubules of *O*. *bicornis* was assessed by quantitative PCR (Fig 4B). CYP9BU1 was found to be highly expressed in the Malpighian tubules, the functional equivalents of vertebrate kidneys, consistent with a primary role in xenobiotic detoxification. In contrast CYP9BU2 was expressed at equivalent levels in the Malpighian tubules, the midgut and the brain (Fig 4B).

## Discussion

The genomes of all bee species sequenced to date have a considerably reduced complement of cytochrome P450s compared to those of other insect species [12,16]. This suggests that, like humans [17], bees may depend on a relatively small subset of generalist P450s for the detoxification of xenobiotics [2]. An emerging body of work on eusocial bees has provided strong support for this hypothesis, with P450s of the CYP9Q subfamily identified as metabolisers of insecticides from three different classes [3,4], and key determinants of honey bee and bumble bee sensitivity to neonicotinoids [3]. In this study we examined the extent to which these findings apply to solitary bees, using the red mason bee, *O*. *bicornis* as a model. Consistent with data from honey bees and bumblebees sequencing of the *O*. *bicornis* genome revealed a reduced P450 inventory in comparison to most other insects, however, in contrast to these species no members of the CYP9Q P450 subfamily were present in the curated CYPome. We interrogated the recently published genomes of several other solitary and eusocial bee species [12] and confirmed that the CYP9Q subfamily is ubiquitous in the CYPome of sequenced social bees (represented by 2–3 genes in most species) but missing in all solitary bee genomes apart from *Habropoda laboriosa*, a species in the family Apidae, which has a single CYP9Q gene (*CYP9Q9*) (S3 Fig). Solitary bees are the ancestral state from which social bees evolved [12] suggesting the CYP9Q subfamily expanded after social bees diverged from solitary bees. A rapid birth–death model of evolution is characteristic of xenobiotic-metabolizing P450s, in contrast to P450s with endogenous functions [18], and the expansion of the CYP9Q subfamily in social bees may have occurred to allow xenobiotics specifically associated with this life history to be detoxified. In relation to this, recent analysis of the CYPomes of ten bee species has suggested that the expansion of the CYP6AS subfamily in perennial eusocial bees resulted from increased exposure to phytochemcials, as a result of the concentration of nectar into honey, pollen into beebread and plant resins into propolis [19].

The finding that most solitary bees lack the CYP9Q subfamily raises important questions about their capacity to metabolise and, by extension tolerate, certain pesticides. Thus, a key finding from our study is that despite the absence of the CYP9Q subfamily *O*. *bicornis* exhibits similar levels of sensitivity to the neonicotinoids imidacloprid and thiacloprid as honey bees and bumblebees, and, like these species, marked tolerance to the latter compound. We show that the observed variation in the sensitivity of *O*. *bicornis* to thiacloprid and imidacloprid does not result from differences in their affinity for the nAChR, or speed of cuticular penetration, but rather variation in their speed/efficiency of metabolism by cytochrome P450s. Functional characterisation revealed that, in the absence of the CYP9Q subfamily, *O*. *bicornis* employs P450s from the CYP9BU subfamily to detoxify the *N*-cyanoamidine neonicotinoid thiacloprid. While the CYP9BU subfamily is currently unique to *O*. *bicornis* phylogeny shows it is more closely related to the CYP9Q subfamily, with which it appears to share a recent common ancestor, than any other bee P450 subfamily. Given that we show that CYP9BU1 appears to be particularly effective in metabolising *N*-cyanoamidine neonicotinoids it will be important to explore which P450s other solitary bee species, such as the economically important leafcutter bee, *Megachile rotundata*, use to detoxify pesticides in the absence of this subfamily (S3 Fig).

Work on other insect species has shown that insecticide-metabolising P450s may be constitutively expressed or induced upon exposure to xenobiotic substrates [20]. We found no evidence of induction of any *O*. *bicornis* P450s in response to exposure to sublethal concentrations of thiacloprid or imidacloprid suggesting that constitutive expression of these enzymes provides protection against neonicotinoids. Their detoxification capacity may be further enhanced by expression in tissues with specialised roles in metabolism/excretion, and it is notable that CYP9BU1 is expressed at particularly high levels in the Malpighian tubules. The overexpression of CYP9BU1 in these osmoregulatory and detoxifying organs is highly consistent with a primary role in xenobiotic metabolism and parallels the high expression of CYP9Q3 in this tissue—the primary metaboliser of neonicotinoids in honey bees [3].

In summary, we show that the solitary bee *O*. *bicornis* is equipped with key biochemical defence enzymes that provide protection against certain insecticides. Together with previous work this demonstrates that while the underlying P450s involved may be different in *O*. *bicornis* and eusocial bees, the overarching detoxification pathways used by these species to metabolise neonicotinoids is conserved. Identification of the P450s responsible for the observed tolerance of *O*. *bicornis* to *N*-cyanoamidine neonicotinoids can be used to support ecotoxicological risk assessment and safeguard the health of this important pollinator. For example, the recombinant enzymes developed in our study can be used to screen existing pesticides to identify and avoid synergistic pesticide-pesticide interactions that inhibit these enzymes [21], and to examine the metabolic liability of future lead compounds as part of efforts to develop pest-selective chemistry. The genomic resources, tools and knowledge generated in this study are particularly timely as *O*. *bicornis* has recently been proposed as a representative solitary bee species for inclusion in future risk assessment of plant protection products in Europe [10].

## Materials and methods

### Sequencing and *de novo* assembly of the *O*. *bicornis* genome

Genomic DNA was extracted from a single male bee using the E.Z.N.A Insect DNA kit (Omega Bio-Tek) following the manufacturer’s protocol. DNA quantity and quality was assessed by spectrophotometry using a NanoDrop (Thermo Scientific), Qubit assay (ThermoFisher) and gel electrophoresis. Sufficient DNA from a single male bee was obtained for the preparation of a single PCR-free paired-end library and 5 long mate pair Nextera libraries that were sequenced on an Illumina HiSeq 2500 using a 250bp read metric at Earlham Institute, Norwich, UK. To improve the quality of subsequent gene prediction RNA sequencing was also performed. For this RNA was extracted from female and male *O*. *bicornis* 24 h after emergence using the Isolate RNA Mini Kit (Bioline) according to the manufacturer’s instructions. The quantity and quality of RNA was checked as described above. RNA was used as a template for the generation of barcoded libraries (TrueSeq RNA library preparation, Illumina) and RNA samples sequenced to high coverage on an Illumina HiSeq2500 flowcell (100 bp paired-end reads). All sequence data have been deposited under NCBI BioProject PRJNA285788.

Reads were assembled using DISCOVAR_*de-novo*–v 52488 [22] using default parameters. All sequences >500 bp from the initial draft assembly were used in scaffolding with 5 Illumina Nextera mate-pair libraries using Redundans–v 0.12a [23] with default parameters. To further increase the contiguity of the draft genome we applied a third scaffolding step, making use of the RNAseq data. Transcriptome contig sequences of *O*. *bicornis* and protein sequences of a closely related species *Megachile rotundata*, were mapped sequentially using L_RNA_scaffolder [24] and PEP_scaffolder [25]. The first round of gene prediction was performed using BRAKER–v 2.1.0 [26] utilising RNAseq data to improve gene calling. To generate training sets for *ab-initio* gene modellers AUGUSTUS [27] and SNAP [28], we searched core eukaryotic and insecta orthologous genes in the *O*. *bicornis* assembly using CEGMA–v 2.5.0 [29] and BUSCO–v 3.0.0 [11] respectively. BUSCO gene models were used to train AUGUSTUS–v 2.5.5, and SNAP (https://github.com/KorfLab/SNAP) was trained using the CEGMA gene models. Another set of hidden markov gene models was generated by GeneMark-ES–v 4.32.0 [30]. In addition, a custom *O*. *bicornis* specific repeat library was built from the assembly using RepeatModeler–v 1.0.4 [31]. To make use of expression data and exploit spliced alignments in genome annotation, expressed transcripts assembled from RNAseq data were further mapped to the *O*. *bicornis* genome using PASA–v 2.3.3 [32]. We initially ran MAKER2 [33] with just the *O*. *bicornis* assembly and EST data, collected from NCBI, followed by three consecutive iterations with the draft genome sequence, transcriptome dataset, models from BRAKER, SNAP and GeneMark-ES, the *O*. *bicornis* specific repeat library and the Swiss-Prot database (accessed at May 23, 2016). Between iterations, the BRAKER and SNAP models were retrained. As BRAKER models are originally predicted from AUGUSTUS, we used AUGUSTUS to train BRAKER models in each successive MAKER2 iteration according to the best-practice MAKER2 workflow. Finally, BRAKER and MAKER2 prediction sets, including PASA alignments, alignment of *M*. *rotundata* proteins using exonerate–v 2.4.0 were combined to generate a non-redundant gene set using EvidenceModeler–v 1.1.1 [34]. The final annotation set for *O*. *bicornis* was compared to other bee genomes to characterize orthology. The proteomes of *Apis mellifera*, *Apis florea*, *Bombus terrestris*, *Bombus impatiens*, *Megachile rotundata*, were downloaded from NCBI, and OrthoFinder–v 1.1.8 [35] was used to define orthologous groups of genes between these peptide sets. P450 sequences were recovered from the bee species using three approaches: 1) Text searches of existing annotation, 2) mining P450 gene sequences from ortholog data generated above, and 3) iterative BLAST searches using *A*. *mellifera* curated P450 genes as queries. All obtained sequences were then manually inspected and curated to generate a final list of P450 genes for each species which were named by the P450 nomenclature committee. Accession numbers are provided in S5 Table.

### Phylogenetic analysis

For phylogenetic analysis, manually curated protein sequences of cytochrome P450 genes were aligned using MUSCLE v3.8.31 [36]. FMO2-like (Protein ID: XP_016772196.1) and CYP315A1 from *A*. *mellifera* were used as an outgroup for phylogenies displayed in Fig 1 and S3 Fig respectively. An initial likelihood phylogenetic tree was created using the R package “phangorn: Phylogenetic Reconstruction and Analysis” v.2.4.0 [37]. Parameters including proportion of variable size (I) and gamma rate (G) were optimized using amino acid substitution matrices JTT for Fig 1 and S3 Fig and LG for S2 Fig based on minimum Bayesian information criterion (S9 Table) [37]. Finally rooted (Fig 1 and S3 Fig) or unrooted (S2 Fig) consensus trees of 1,000x bootstrapping using nearest-neighbor interchange were created and visualized using the R package “ggtree” v1.12.0 [37,38].

### Acute contact toxicity bioassays

*O*. *bicornis* cocoons were purchased from Dr Schubert Plant Breeding (Landsberg, Germany) and stored at 4°C in constant darkness. To trigger emergence cocoons were transferred to an incubator (25°C, 55% RH, L16:D8) with emerged bees fed *ab libitum* with Biogluc (62% sugar concentration consisting of 37.5% fructose, 34.5% glucose, 25% sucrose, 2% maltose, and 1% oligosaccharides) (Biobest), soaked into a piece of cotton wool inside a plastic dish. Males (which are usually first to emerge) were removed from cages and discarded to reduce any unnecessary stress to the females used in insecticide bioassays.

Acute contact toxicity bioassays on unmated 2 day old female *O*. *bicornis* were conducted following the OECD Honey Bee Test guidelines, with modification where necessary [39]. Bees were anaesthetised with CO_2_ for 5–10 seconds to allow application of insecticide. 1 μL of technical grade imidacloprid was applied to the dorsal thorax of each bee at concentrations of 0.0001, 0.001, 0.01, 0.1, 1, and 10 μg/μL. No mortality was observed using the same concentrations of thiacloprid so a limit test of 100 μg/bee was performed. Control bees were treated with 1 μL 100% acetone. Three replicates of 10 bees were tested for each concentration. Tested individuals were placed back into cages in the incubator (25°C, 55% RH, L16:D8), with five bees per cage. In piperonyl butoxide (PBO) synergist bioassays, bees were first treated with the maximum sublethal dose (in this case 100 μg/μL) of PBO followed by insecticide one hour later. Synergist bioassays included an additional control group treated only with PBO. Mortality was assessed 48 and 72 hours after application. Probit analysis was used to calculate the LD_50_ values, slope, and synergism ratio (where relevant) for each insecticide (Genstat v.18 (VSNI 2015)).

### Radioligand competition binding studies

[^3^H]imidacloprid (specific activity 1.406 GBq μmol^−1^) displacement studies were conducted using membrane preparations isolated from frozen (−80°C) *O*. *bicornis* heads, following previously published protocols [13]. Briefly, bee heads weighing 10 g were homogenized in 200 ml ice-cold 0.1 M potassium phosphate buffer, pH 7.4 containing 95 mM sucrose using a motor-driven Ultra Turrax blender. The homogenate was then centrifuged for 10 min at 1200 g and the resulting supernatant filtered through five layers of cheesecloth with protein concentration determined using Bradford reagent (Sigma) and bovine serum albumin (BSA) as a reference. Assays were performed in a 96-well microtitre plate with bonded GF/C filter membrane (Packard UniFilter-96, GF/C) and consisted of 200 μl of homogenate (0.48 mg protein), 25 μl of [^3^H]imidacloprid (576 pM) and 25 μl of competing ligand. Ligand concentrations used ranged from 0.001 to 10 000 nM and were tested in triplicate per competition assay. The assay was started by the addition of homogenate and incubated for 60 min at room temperature. Bound [^3^H]imidacloprid was quantified by filtration into a second 96-well filter plate (conditioned with ice-cold 100 mM potassium phosphate buffer, pH 7.4 (including BSA 5 g litre−1)) using a commercial cell harvester (Brandel). After three washing steps (1 ml each) with buffer the 96-well filter plates were dried overnight. Each well was then loaded with 25 μl of scintillation cocktail (Microszint-O-Filtercount, Packard) and the plate counted in a Topcount scintillation counter (Packard). Non-specific binding was determined using a final concentration of 10 μM unlabelled imidacloprid. All binding experiments were repeated twice using three replicates per tested ligand concentration. Data were analysed using a 4 parameter logistic non-linear fitting routine (GraphPad Prism version 7 (GraphPad Software, CA, USA)) in order to calculate IC_50_-values (concentration of unlabelled ligand displacing 50% of [^3^H]imidacloprid from its binding site). Non-linear regression model fitting and statistical comparison of the slopes obtained was performed in the *drc* package in R [40].

### Pharmacokinetic studies

Bees were anaesthetised with CO_2_ for 5–10 seconds to allow application of insecticide. 5,000 ppm of [^14^C]imidacloprid or [^14^C]thiacloprid was applied to the dorsal thorax of each bee using a Hamilton repeating dispenser. Three replicates of five bees were placed into cages and fed a 50% sucrose solution from vertically hanging 2 ml syringes. Control bees were treated with acetone. Radiolabelled insecticide was rinsed off of each group of bees at set time intervals (0, 2, 4 and 24 hours after application) with acetonitrile water (90:10) three times. The acetone-washed bees were then individually combusted at 900°C in an Ox 120c oxidizer (Harvey Instruments Co., USA) followed by liquid scintillation counting of the released ^14^CO_2_ in an alkaline scintillation cocktail (Ultima Gold, PerkinElmer) using a liquid scintillation analyser (Perkin Elmer Tri-Carb 2910 TR). The levels of excreted [^14^C]imidacloprid or [^14^C]thiacloprid, and/or metabolites, were measured by wiping cages with filter papers dipped in acetone and 0.5 mL aliquots of cuticular rinse or filter papers were added to 3 mL of scintillation fluid cocktail and the radioactivity was quantified by liquid scintillation analysis as above. An unpaired t-test was used to compare the penetration of the two compounds at each time point.

### PCR validation of candidate P450s

Sequences of *O*. *bicornis* candidate genes were verified by PCR as follows: Adult female *O*. *bicornis* were flash frozen in liquid nitrogen and stored at -80°C prior to extractions. RNA was extracted from a pool of 3–5 bees using the RNeasy Plus kit (QIAGEN) following the manufacturer’s protocol. The quantity and quality of RNA were assessed as described above. First-strand cDNA was synthesised at a concentration of 200 ng/μL by reverse transcription using SuperScript III Reverse Transcriptase (Invitrogen) according to the manufacturer’s protocol. 25μL reactions contained 1.5U DreamTaq DNA Polymerase (Thermofisher), 10mM of forward and reverse primers (S10 Table) and 200 ng of cDNA. PCR reaction temperature cycling conditions were 95°C for 2 minutes, followed by 35 cycles of 95°C for 20 seconds (denaturation), 60°C for 20 seconds (annealing), and 72°C for 7.5 minutes (elongation). PCR products were visualised on a 1% agarose gel and purified using QIAquick PCR purification kit (QIAGEN). Samples were sequenced by Eurofins (Eurofins Scientific group, Belgium) and analysed using Geneious v8.1.3 software (Biomatters Ltd, New Zealand).

### Functional expression of candidate P450s

*O*. *bicornis* P450 genes and house fly NADPH-dependent cytochrome P450 reductase (CPR) (GenBank accession no. Q07994) genes were codon optimised for expression in lepidopteran cell lines, synthesized (Geneart, CA, USA) and inserted into the pDEST8 expression vector (Invitrogen). The PFastbac1 vector with no inserted DNA was used to produce a control virus. The recombinant baculovirus DNA was constructed and transfected into *Trichoplusia ni* (High five cells, Thermo Fisher) using the Bac-to-Bac baculovirus expression system (Invitrogen) according to the manufacturer’s instructions. The titre of the recombinant virus was determined following protocols of the supplier. High Five cells grown to a density of 2 x 10^6^ cells ml^-1^ were co-infected with recombinant baculoviruses containing each bee P450 and CPR with a range of MOI (multiplicity of infection) ratios to identify the optimal conditions. Control cells were co-infected with the baculovirus containing vector with no insert (ctrl-virus) and the recombinant baculovirus expressing CPR using the same MOI ratios. Ferric citrate and δ-aminolevulinic acid hydrochloride were added to a final concentration of 0.1 mM at the time of infection and 24 h after infection to compensate the low levels of endogenous heme in the insect cells. After 48 h, cells were harvested, washed with PBS, and microsomes of the membrane fraction prepared according to standard procedures and stored at −80°C [41]. Briefly, pellets were homogenised for 30 s in 0.1 M Na/K-phosphate buffer, pH 7.4 containing 1 mM EDTA and DTT and 200 mM sucrose using a Fastprep (MP Biomedicals), filtered through miracloth and centrifuged for 10 min at 680g at 4°C. The supernatant was then centrifuged for 1 h at 100,000g at 4°C, with the pellet subsequently resuspended in 0.1M Na/K-phosphate buffer pH 7.6 containing 1 mM EDTA and DTT and 10% glycerol using a Dounce tissue grinder. P450 expression and functionality was estimated by measuring CO-difference spectra in reduced samples using a Specord 200 Plus Spectrophotometer (Analytik Jena) and scanning from 500 nm to 400 nm [41]. The protein content of samples was determined using Bradford reagent (Sigma) and bovine serum albumin (BSA) as a reference.

### Metabolism assays and UPLC-MS/MS analysis

Metabolism of thiacloprid and imidacloprid was assessed by incubating recombinant P450/CPR (5 pmol/well) or control virus/CPR (5 pmol/well) with each insecticide (25 μM) in the presence of an NADPH regeneration system at 30±1°C, shaking, for 1 hour. Three replicates were performed for each data point and the total assay volume was 200 μL. Samples incubated without NADPH served as a control. The reactions were terminated by the addition of ice-cold acetonitrile (to 80% final concentration), centrifuged for 10 min at 3000 g and the supernatant analyzed by tandem mass spectrometry as described previously [42]. LC-MS/MS analysis was performed on a Waters Acquity UPLC coupled to a Sciex API 4000 mass spectrometer and an Agilent Infinity II UHPLC coupled to a Sciex QTRAP 6500 mass spectrometer utilizing electrospray ionization. For the chromatography on a Waters Acquity HSS T3 column (2.1x50 mm, 1.8 μm), acetonitrile/water/0.1% formic acid was used as the eluent in gradient mode. For detection and quantification in positive ion mode, the MRM transitions 253 > 186, 269 > 202 (thiacloprid, OH-thiacloprid), and 256 > 175, 272 > 191 (imidacloprid, OH-imidacloprid) were monitored. The peak integrals were calibrated externally against a standard calibration curve. Recovery rates of parent compounds using microsomal fractions without NADPH were normally close to 100%. Substrate turnover was determined using GraphPad Prism version 7 (GraphPad Software, CA, USA).

### Transgenic expression of *CYP9BU1* and *CYP9BU2* in *Drosophila melanogaster*

*CYP9BU1* and *CYP9BU2* were codon optimised for *D*. *melanogaster* expression and cloned into the pUASTattB plasmid (GenBank: EF362409.1). These constructs were used to create transgenic fly lines, which were then tested in insecticide bioassays against imidacloprid and thiacloprid, as described previously [3].

### P450 expression studies

To examine if P450 expression in *O. bicornis* is induced by exposure to sublethal concentrations of neonicotinoids, imidacloprid and thiacloprid were dissolved in acetone to the highest concentration possible, before being diluted to the LD_10_ of imidacloprid (0.0001 μg/bee) and thiacloprid (0.01 μg/bee) with 50% sucrose (w/v) in order to limit the amount of acetone consumed by bees. Prior to commencing oral bioassays bees underwent a 24 hour ‘training’ period in the Nicot cages to enable them to learn to feed from the syringes. This was followed by a 16h starvation period to encourage subsequent feeding. 15μL of the insecticide/sucrose solution was supplied orally to the bees in disposable plastic syringes. Control bees were fed 15μL of a sucrose solution containing the same volume of acetone used to make up the insecticide/sucrose solutions. When all of the solution had been consumed the bees were fed *ab libitum* with a 50% sucrose solution for 24 h. After this period for each condition four replicates comprising 5 bees per replicate were snap frozen in liquid nitrogen and RNA extracted from each replicate as above. RNA was used as a template for the generation of barcoded libraries (TrueSeq RNA library preparation, Illumina) which were sequenced across two lanes of an Illumina HiSeq2500 flowcell (100 bp paired end reads). Sequencing was carried out by Earlham Institute, Norwich, UK. To identify genes differentially expressed between control and the treatment the Tuxedo workflow was used to map with TopHat against the annotated reference genome, to estimate gene expression with Cufflinks and test for differential expression with Cuffdiff [43].

To examine the expression of candidate P450 genes in tissues with a known role in detoxification or the site of insecticide action the brain, midgut and Malpighian tubules were extracted from flash frozen adult female *O*. *bicornis*. RNA*later*-ICE (Life technologies) was used to preserve RNA during dissections. RNA was extracted as above and first-strand cDNA synthesised using SuperScript III Reverse Transcriptase (Invitrogen) according to the manufacturer’s protocol. Quantitative RT-PCR was carried out using a Rotor Gene 6000 machine with the thermocycling conditions: 3 minutes at 95°C followed by 40 cycles of 95°C for 20 seconds (denaturation), 60°C for 20 seconds (annealing), and 72°C for 7.5 minutes (elongation). A final melt-curve step was included to rule out any non-specific amplification. 15μL reactions consisted of 6μL cDNA (10ng), 7μL of SYBR Green Master Mix (Thermofisher Scientific) and 0.25μM of the forward and reverse primers. All primers were designed using the Prime3 primer design tool (http://biotools.umassmed.edu/bioapps/primer3_www.cgi) and are listed in S10 Table. All primers were designed to amplify a ~200bp region of each target gene with low percentage identity to other target genes. The efficiency of each primer set was examined using a standard curve (concentrations 100–0.01ng of cDNA). Elongation Factor α1 and elongation factor **γ**1 were used as housekeeping genes as these were found to exhibit stable expression between different tissues. Each data point consisted of three technical replicates and four biological replicates. Data were analysed using the _ΔΔ_CT method [44] using the geometric mean of the two housekeeping genes to normalise data.

## Supporting information

S1 Fig Functional annotation of *O*. *bicornis* gene models. (A) Number of genes returning BLAST hits (BLAST), Interpro hits (IP), GO category hits (GO), evidence codes (EC) and KEGG hits (KEGG). (B) Percentage of genes with and without BLAST hits and number of genes correctly mapped after combining evidence from BLAST and GO-Slim. (C) Species distribution of BLAST hits against *O*. *bicornis* protein coding genes.
(TIF)

S2 Fig Unrooted maximum likelihood consensus phylogenetic tree of *O*. *bicornis* P450 genes. Genes are coloured according to their adscription to different P450 clades (CYP2: Blue; CYP3: Red; CYP4: Green; Mitochondrial: Purple).
(TIF)

S3 Fig Rooted maximum likelihood consensus phylogenetic tree of the CYP9 family of P450 gene in 12 bee species. Members of the CYP9Q and CYP9B subfamilies are highlighted using red circles.
(TIF)

S4 Fig K-mer distribution plot generated, from short-read sequencing data, using Genomescope (http://qb.cshl.edu/genomescope/). (TIF)

S5 Fig Distribution plot of repetitive DNA in the *Osmia bicornis* genome. LTR: Long terminal repeat families; LINE: Long interspersed nuclear elements; RC: Rolling circle/Helitron family.
(TIF)

S6 Fig Workflow used for gene modelling and functional annotation. (TIF)

S1 Table Characteristics of the *O*. *bicornis* genome derived from k-mer distribution. (DOCX)

S2 Table Summary statistics of the final *O*. *bicornis* (-v3) genome assembly. (DOCX)

S3 Table Summary of gene features from the *O*. *bicornis*–v3 genome assembly. (DOCX)

S4 Table Benchmarking Universal Single-Copy Orthologs (BUSCO) analysis of the *O*. *bicornis*–v3 genome assembly. (DOCX)

S5 Table Names and accession numbers of curated P450s genes derived from the genome sequences of solitary and social bee species. (DOCX)

S6 Table Log-dose probit mortality data for imidacloprid and thiacloprid against transgenic Drosophila strains expressing *O*. *bicornis* P450 genes. (DOCX)

S7 Table Genes identified as significantly differentially expressed in RNAseq data between imidacloprid treated and untreated *O*. *bicornis* female bees. (DOCX)

S8 Table Genes identified as significantly differentially expressed in RNAseq data between thiacloprid treated and untreated *O*. *bicornis* female bees. (DOCX)

S9 Table Bayesian information criterion (BIC) for data sets of phylogenetic trees using different substitution matrices and parameter optimizations. (DOCX)

S10 Table Sequences of the oligonucleotide primers used in this study. (DOCX)

S11 Table Summary of short read (PE) and long-insert (MP) sequencing data generated in this study. (DOCX)

S12 Table Summary of mate-pair (MP) sequence data generated in this study. (DOCX)

S13 Table Summary statistics of all PE and MP sequencing data generated in this study. (DOCX)

S14 Table Assembly properties at different stages of the *O*. *bicornis* assembly pipeline. (DOCX)

S15 Table Remapping statistics of PE and MP data mapped to the *O*. *bicornis*–v3 assembly. (DOCX)

S16 Table Core Eukaryotic Genes Mapping Approach (CEGMA) analysis of the *O*. *bicornis* genome assembly. (DOCX)

S17 Table Summary statistics derived from ortholog analysis. (DOCX)

S18 Table Per species summary of ortholog analysis. AF: *Apis florea*, AM: *Apis mellifera*, BI: *Bombus impatiens*, BT: *Bombus terrestris*, MR: *Megachile rotundata*, OB: *Osmia bicornis*.
(DOCX)

S19 Table Trinity assembly statistics. (DOCX)

S1 Text Supplementary methods. (DOCX)
