# Supplementary figures and images for: Genomic insights into neonicotinoid sensitivity in the solitary bee Osmia bicornis
Source: PLoS Genet. 2019 Feb 4;15(2):e1007903. doi: 10.1371/journal.pgen.1007903 (PMC6375640; doi:10.1371/journal.pgen.1007903)

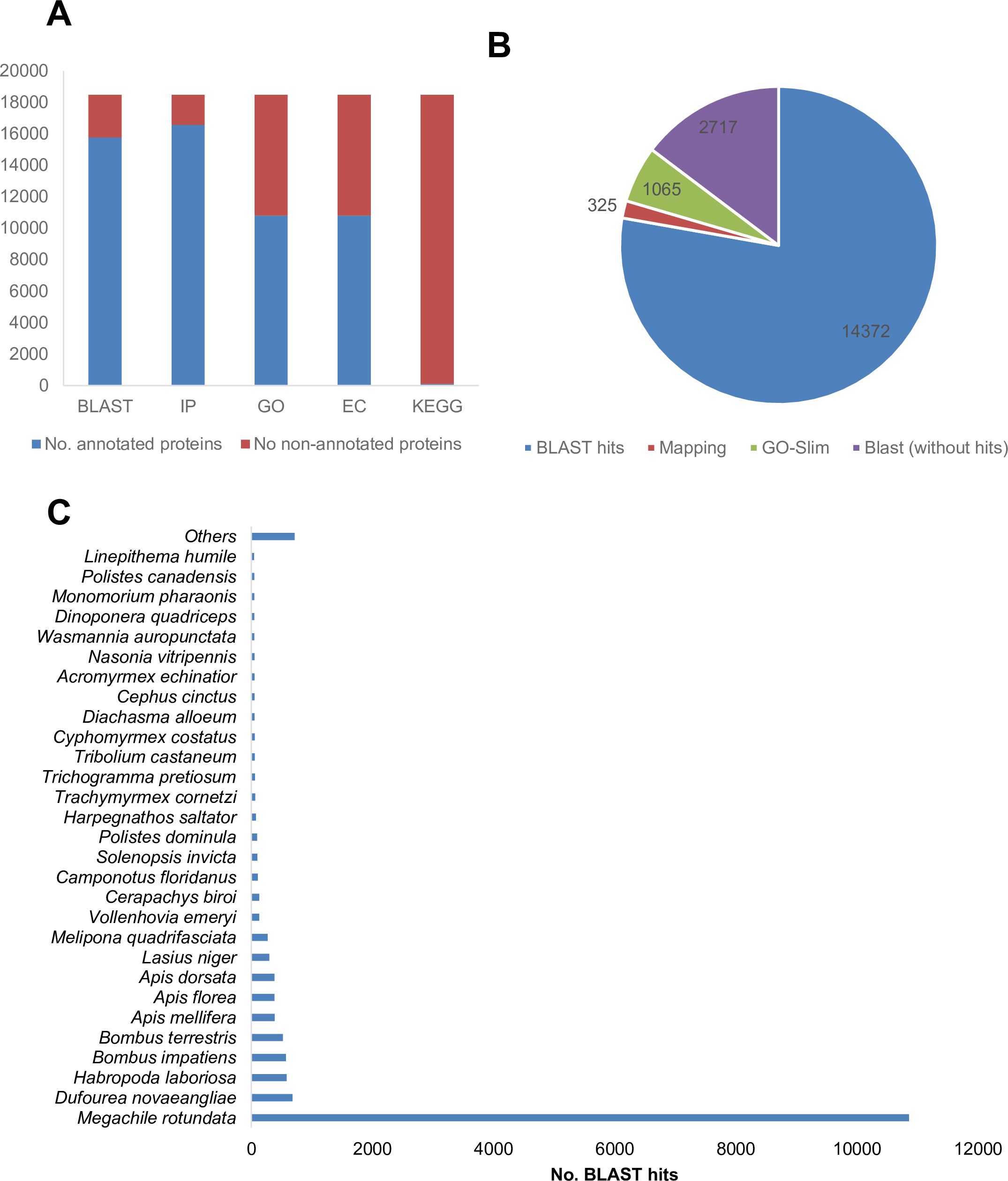

Supplement: S1 Fig — (A) Number of genes returning BLAST hits (BLAST), Interpro hits (IP), GO category hits (GO), evidence codes (EC) and KEGG hits (KEGG). (B) Percentage of genes with and without BLAST hits and number of genes correctly mapped after combining evidence from BLAST and GO-Slim. (C) Species distribution of BLAST hits against O. bicornis protein coding genes. (TIF) [file pgen.1007903.s001.tif]

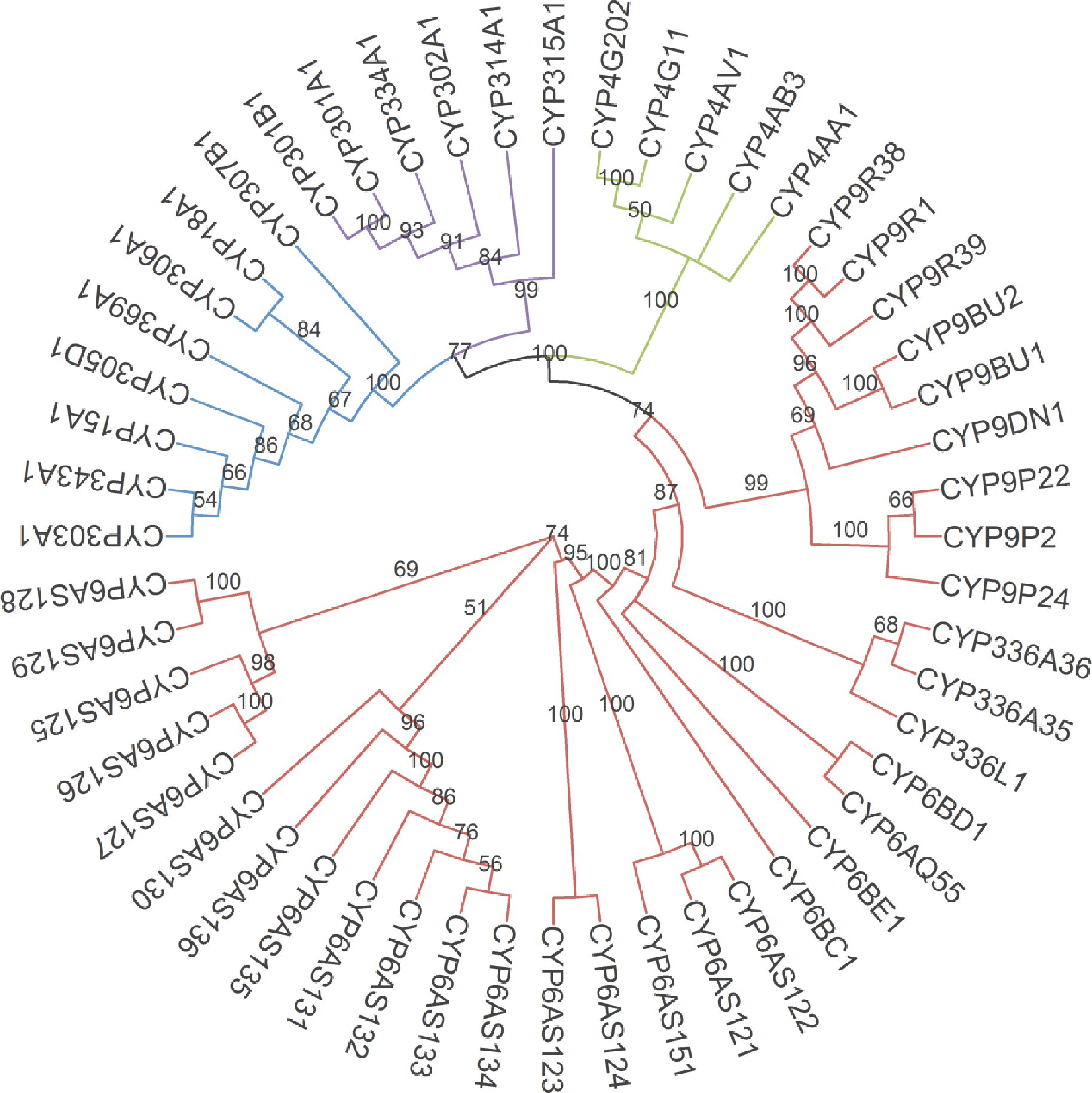

Supplement: S2 Fig — Genes are coloured according to their adscription to different P450 clades (CYP2: Blue; CYP3: Red; CYP4: Green; Mitochondrial: Purple). (TIF) [file pgen.1007903.s002.tif]

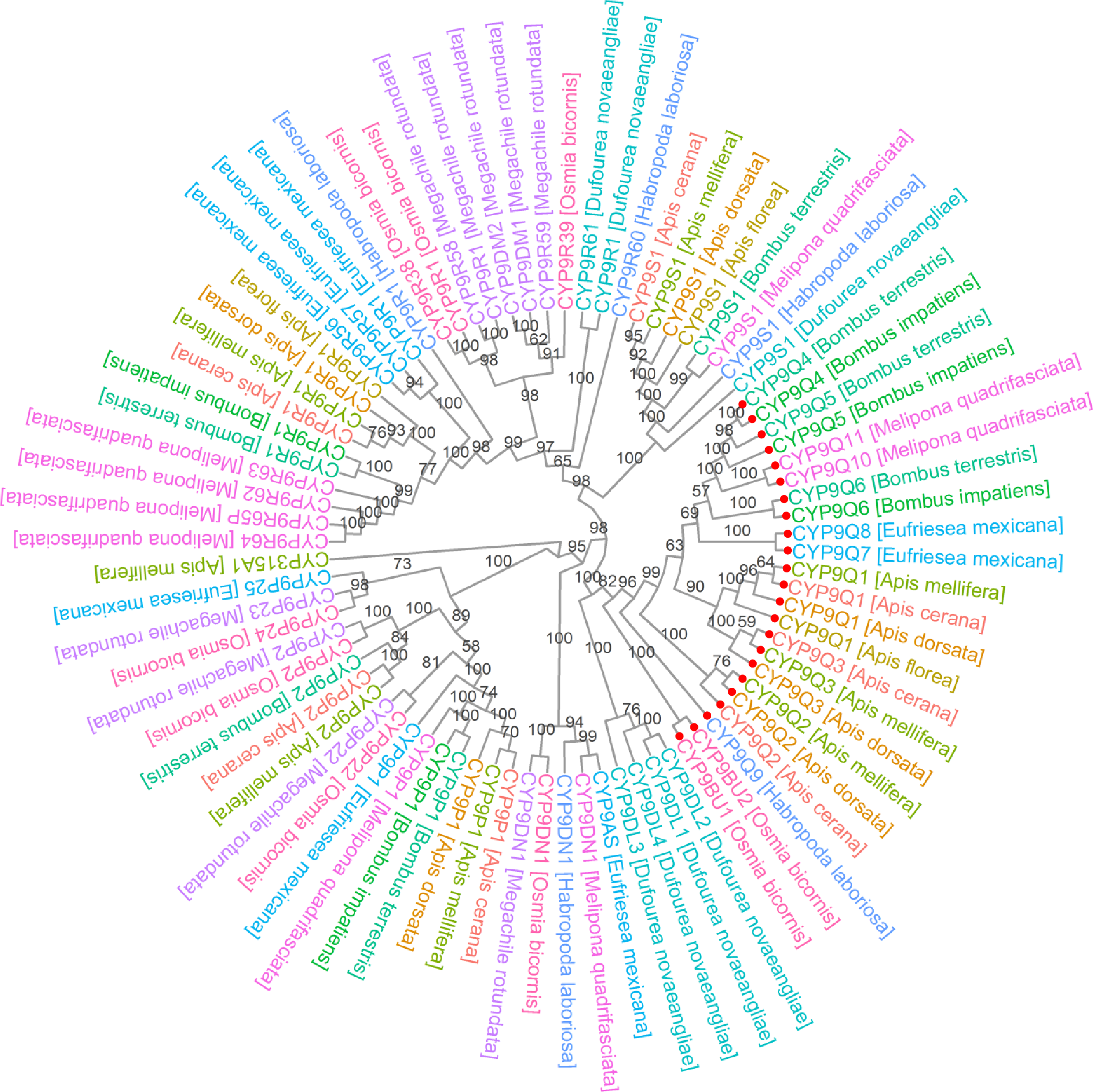

Supplement: S3 Fig — Members of the CYP9Q and CYP9B subfamilies are highlighted using red circles. (TIF) [file pgen.1007903.s003.tif]

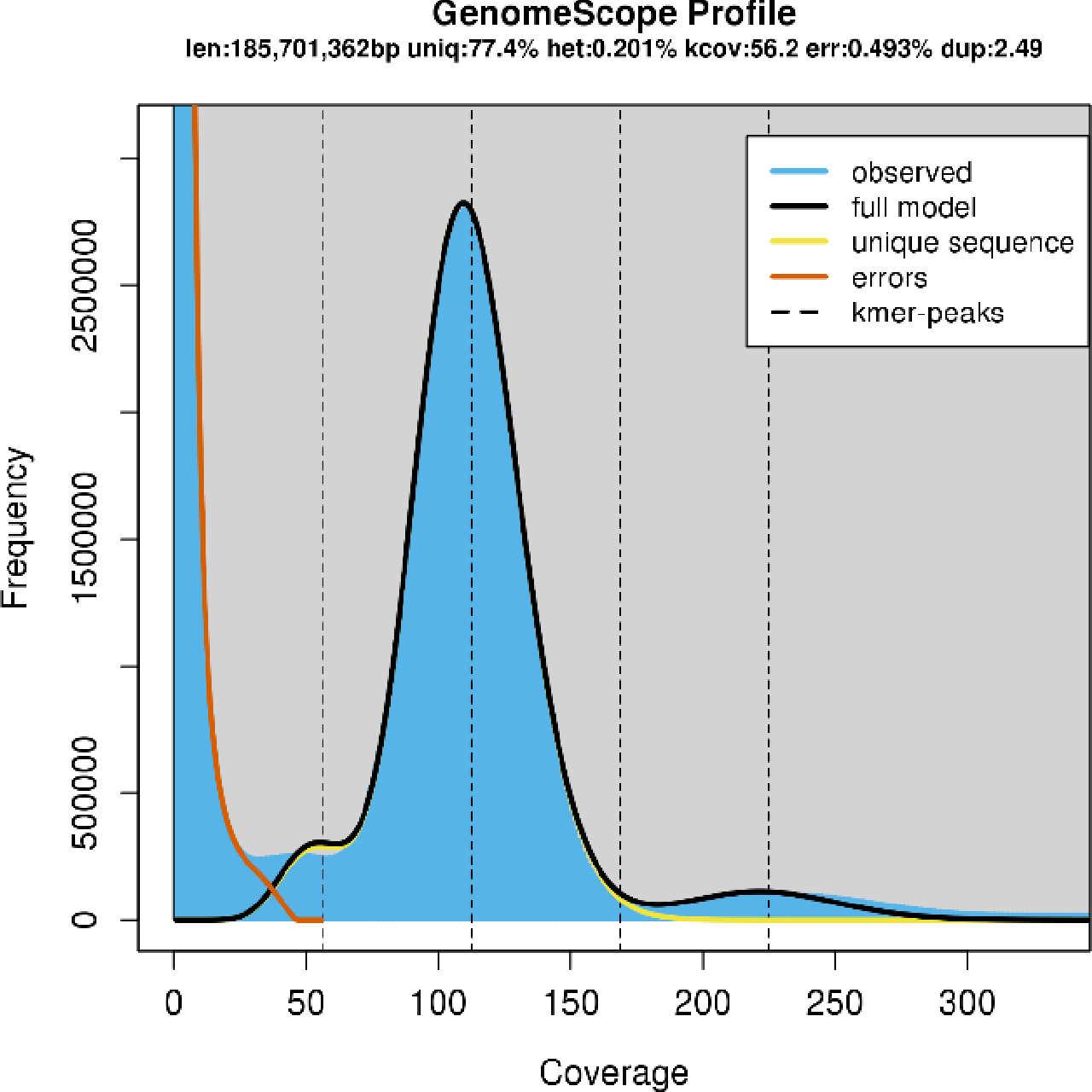

Supplement: S4 Fig — (TIF) [file pgen.1007903.s004.tif]

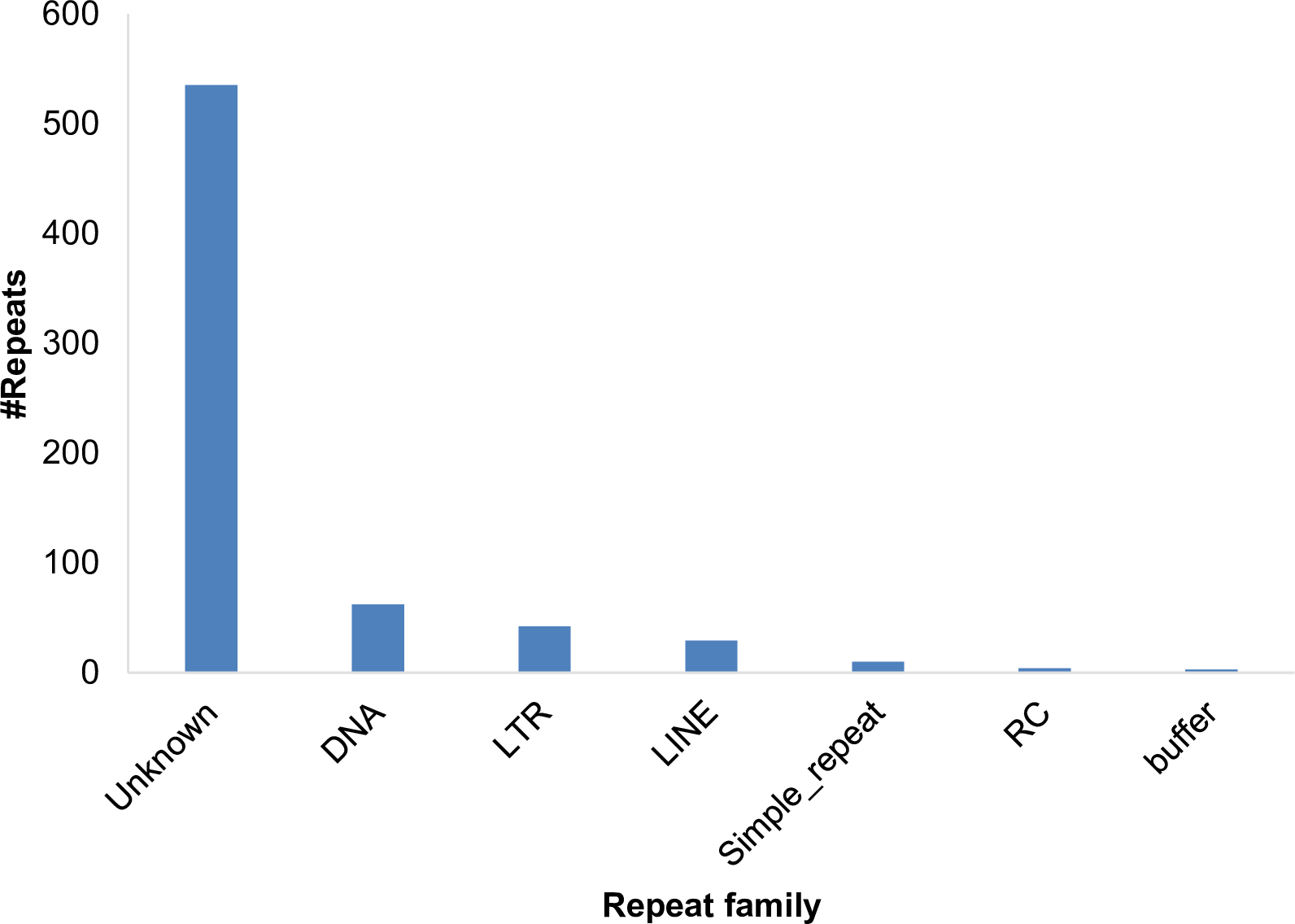

Supplement: S5 Fig — LTR: Long terminal repeat families; LINE: Long interspersed nuclear elements; RC: Rolling circle/Helitron family. (TIF) [file pgen.1007903.s005.tif]

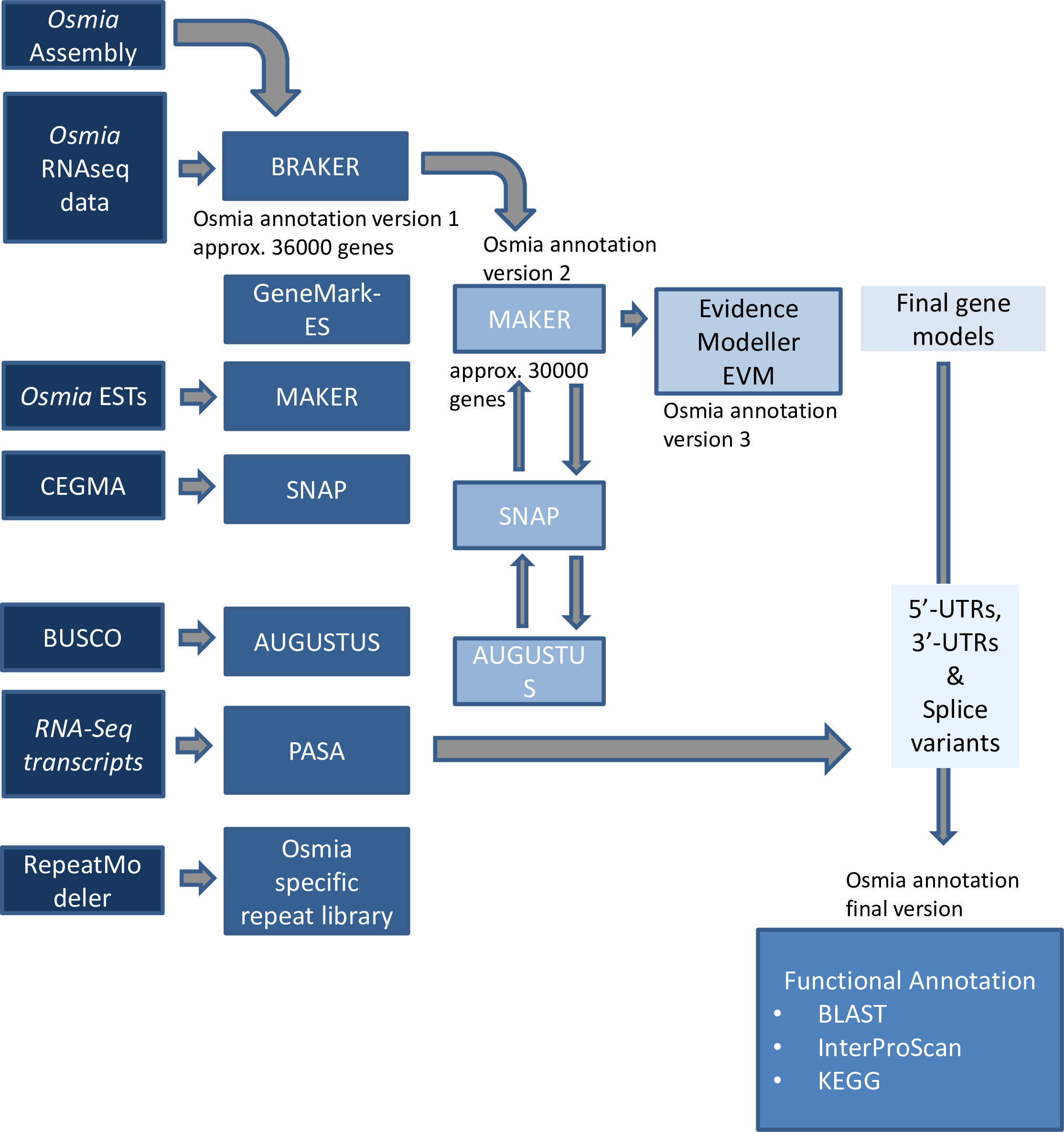

Supplement: S6 Fig — (TIF) [file pgen.1007903.s006.tif]
