# Supplementary material for: Genomic insights into neonicotinoid sensitivity in the solitary bee Osmia bicornis
Source: PLoS Genet. 2019 Feb 4;15(2):e1007903. doi: 10.1371/journal.pgen.1007903 (PMC6375640; doi:10.1371/journal.pgen.1007903)
Supplement: S1 Table — (DOCX) [file pgen.1007903.s007.docx]

| **Property** | **Minimum** | **Maximum** |
| --- | --- | --- |
| Heterozygosity | 0.194% | 0.207% |
| Genome Haploid length | ~185MB | ~186MB |
| Genome Repeat Length | ~42MB | ~42MB |
| Genome Unique Length | 143.5MB | 145.7MB |
| Read Error rate | 0.49% | 0.49% |
