# Supplementary material for: Genomic insights into neonicotinoid sensitivity in the solitary bee Osmia bicornis
Source: PLoS Genet. 2019 Feb 4;15(2):e1007903. doi: 10.1371/journal.pgen.1007903 (PMC6375640; doi:10.1371/journal.pgen.1007903)
Supplement: S2 Table — (DOCX) [file pgen.1007903.s008.docx]

| **Property** | **Values** |
| --- | --- |
| Total number of scaffolds | 12577 |
| Total length | 212931040 |
| Largest scaffold | 4482317 |
| GC (%) | 39.77 |
| N50 | 604175 |
| N75 | 59276 |
| L50 | 97 |
| L75 | 314 |
| Total number of scaffolds (>= 0 bp) | 14071 |
| Total number of scaffolds (>= 1000 bp) | 10223 |
| Total number of scaffolds (>= 5000 bp) | 3141 |
| Total number of scaffolds (>= 10000 bp) | 1534 |
| Total number of scaffolds (>= 25000 bp) | 532 |
| Total number of scaffolds (>= 50000 bp) | 336 |
| Total length (>= 0 bp) | 212931040 |
| Total length (>= 1000 bp) | 210598924 |
| Total length (>= 5000 bp) | 193320581 |
| Total length (>= 10000 bp) | 182023389 |
| Total length (>= 25000 bp) | 166978309 |
| Total length (>= 50000 bp) | 160497702 |
| Number of N's per 100 kbp | 15.79 |
