# Supplementary material for: Genomic insights into neonicotinoid sensitivity in the solitary bee Osmia bicornis
Source: PLoS Genet. 2019 Feb 4;15(2):e1007903. doi: 10.1371/journal.pgen.1007903 (PMC6375640; doi:10.1371/journal.pgen.1007903)
Supplement: S3 Table — (DOCX) [file pgen.1007903.s009.docx]

| **Property** | **Value** |
| --- | --- |
| Total sequence length | 212931040 |
| Number of genes | 14858 |
| Number of mRNAs | 18479 |
| Number of exons | 110523 |
| Number of introns | 92044 |
| Number of CDS | 18479 |
| Total gene length | 77239236 |
| Total mRNA length | 123999314 |
| Total exon length | 42588032 |
| Total intron length | 81595370 |
| Total CDS length | 28207087 |
| Shortest gene | 150 |
| Shortest mRNA | 150 |
| Shortest exon | 1 |
| Shortest intron | 20 |
| Shortest CDS | 141 |
| Longest gene | 393458 |
| Longest mRNA | 393458 |
| Longest exon | 18571 |
| Longest intron | 303153 |
| Longest CDS | 55014 |
| mean gene length | 5198 |
| mean mRNA length | 6710 |
| mean exon length | 385 |
| mean intron length | 886 |
| mean CDS length | 1526 |
| % of genome covered by genes | 36.3 |
| % of genome covered by CDS | 13.2 |
| mean mRNAs per gene | 1 |
| mean exons per mRNA | 6 |
| mean introns per mRNA | 5 |
