# Supplementary material for: Genomic insights into neonicotinoid sensitivity in the solitary bee Osmia bicornis
Source: PLoS Genet. 2019 Feb 4;15(2):e1007903. doi: 10.1371/journal.pgen.1007903 (PMC6375640; doi:10.1371/journal.pgen.1007903)
Supplement: S4 Table — (DOCX) [file pgen.1007903.s010.docx]

| **Clade** | **% BUSCO** | **#BUSCO** | **Complete BUSCO** | **Single copy** | **Duplicated** | **Fragmented** | **Missing** |
| --- | --- | --- | --- | --- | --- | --- | --- |
| Eukaryota | 99.4 | 303 | 301 | 296 | 5 | 0 | 2 |
| Arthropoda | 99.9 | 1066 | 1065 | 1059 | 6 | 1 | 0 |
| Insecta | 99.7 | 1658 | 1652 | 1646 | 6 | 4 | 2 |
