# Supplementary material for: Genomic insights into neonicotinoid sensitivity in the solitary bee Osmia bicornis
Source: PLoS Genet. 2019 Feb 4;15(2):e1007903. doi: 10.1371/journal.pgen.1007903 (PMC6375640; doi:10.1371/journal.pgen.1007903)
Supplement: S5 Table — (DOCX) [file pgen.1007903.s011.docx]

| **Species** | **Accession number** | **CYP name** |
| --- | --- | --- |
| Osmia_bicornis | MH500618 | CYP4G11 |
| Osmia_bicornis | MH500613 | CYP4G202 |
| Osmia_bicornis | MH500648 | CYP4AA1 |
| Osmia_bicornis | MH500632 | CYP4AB3 |
| Osmia_bicornis | MH500612 | CYP4AV1 |
| Osmia_bicornis | MH500625 | CYP6AQ55 |
| Osmia_bicornis | MH500627 | CYP6AS121 |
| Osmia_bicornis | MH500639 | CYP6AS122 |
| Osmia_bicornis | MH500644 | CYP6AS123 |
| Osmia_bicornis | MH500646 | CYP6AS124 |
| Osmia_bicornis | MH500622 | CYP6AS125 |
| Osmia_bicornis | MH500614 | CYP6AS126 |
| Osmia_bicornis | MH500637 | CYP6AS127 |
| Osmia_bicornis | MH500640 | CYP6AS128 |
| Osmia_bicornis | MH500642 | CYP6AS129 |
| Osmia_bicornis | MH500641 | CYP6AS130 |
| Osmia_bicornis | MH500655 | CYP6AS131 |
| Osmia_bicornis | MH500653 | CYP6AS132 |
| Osmia_bicornis | MH500610 | CYP6AS133 |
| Osmia_bicornis | MH500654 | CYP6AS134 |
| Osmia_bicornis | MH500611 | CYP6AS135 |
| Osmia_bicornis | MH500643 | CYP6AS136 |
| Osmia_bicornis | MH500615 | CYP6AS151 |
| Osmia_bicornis | MH500635 | CYP6BC1 |
| Osmia_bicornis | MH500651 | CYP6BD1 |
| Osmia_bicornis | MH500616 | CYP6BE1 |
| Osmia_bicornis | MH500623 | CYP9DN1 |
| Osmia_bicornis | MH500628 | CYP9P2 |
| Osmia_bicornis | MH500630 | CYP9P22 |
| Osmia_bicornis | MH500629 | CYP9P24 |
| Osmia_bicornis | MH500638 | CYP15A1 |
| Osmia_bicornis | MH500621 | CYP18A1 |
| Osmia_bicornis | MH500645 | CYP301A1 |
| Osmia_bicornis | MH500624 | CYP301B1 |
| Osmia_bicornis | MH500633 | CYP302A1 |
| Osmia_bicornis | MH500649 | CYP303A1 |
| Osmia_bicornis | MH500631 | CYP305D1 |
| Osmia_bicornis | MH500650 | CYP306A1 |
| Osmia_bicornis | MH500620 | CYP307B1 |
| Osmia_bicornis | MH500652 | CYP314A1 |
| Osmia_bicornis | MH500609 | CYP315A1 |
| Osmia_bicornis | MH500647 | CYP334A1 |
| Osmia_bicornis | MH500619 | CYP336A35 |
| Osmia_bicornis | MH500617 | CYP336A36 |
| Osmia_bicornis | MH500626 | CYP336L1 |
| Osmia_bicornis | MH500634 | CYP343A1 |
| Osmia_bicornis | MH500636 | CYP369A1 |
| Osmia_bicornis | MH500604 | CYP9BU1 |
| Osmia_bicornis | MH500605 | CYP9BU2 |
| Osmia_bicornis | MH500608 | CYP9R1 |
| Osmia_bicornis | MH500606 | CYP9R38 |
| Osmia_bicornis | MH500607 | CYP9R39 |
| Apis_mellifera | tr\|Q306I1\|Q306I1_APIME | CYP4G11 |
| Apis_mellifera | tr\|A0A088A7F3\|A0A088A7F3_APIME | CYP4AB3 |
| Apis_mellifera | tr\|A0A087ZZZ6\|A0A087ZZZ6_APIME | CYP4AV1 |
| Apis_mellifera | tr\|F1DPP0\|F1DPP0_APIME | CYP6AQ1 |
| Apis_mellifera | tr\|A0A087ZNI4\|A0A087ZNI4_APIME | CYP6AS1 |
| Apis_mellifera | tr\|A0A088AEK0\|A0A088AEK0_APIME | CYP6AS2 |
| Apis_mellifera | tr\|A0A088AEK1\|A0A088AEK1_APIME | CYP6AS3 |
| Apis_mellifera | tr\|A0A088AEJ9\|A0A088AEJ9_APIME | CYP6AS4 |
| Apis_mellifera | tr\|Q309A5\|Q309A5_APIME | CYP6AS5 |
| Apis_mellifera | tr\|A0A088AEK4\|A0A088AEK4_APIME | CYP6AS5P |
| Apis_mellifera | tr\|A0A088AEK7\|A0A088AEK7_APIME | CYP6AS7 |
| Apis_mellifera | tr\|A0A088AEJ2\|A0A088AEJ2_APIME | CYP6AS8 |
| Apis_mellifera | tr\|A0A087ZNI0\|A0A087ZNI0_APIME | CYP6AS10 |
| Apis_mellifera | tr\|A0A088AEJ0\|A0A088AEJ0_APIME | CYP6AS11 |
| Apis_mellifera | tr\|A0A088AEK2\|A0A088AEK2_APIME | CYP6AS12 |
| Apis_mellifera | tr\|A0A087ZNI3\|A0A087ZNI3_APIME | CYP6AS13 |
| Apis_mellifera | tr\|A0A088AEI9\|A0A088AEI9_APIME | CYP6AS14 |
| Apis_mellifera | tr\|A0A088A3A5\|A0A088A3A5_APIME | CYP6AS15 |
| Apis_mellifera | tr\|A0A087ZNI2\|A0A087ZNI2_APIME | CYP6AS16 |
| Apis_mellifera | tr\|A0A088AEK6\|A0A088AEK6_APIME | CYP6AS17 |
| Apis_mellifera | tr\|A0A088AEK5\|A0A088AEK5_APIME | CYP6AS18 |
| Apis_mellifera | gnl\|Amel_4.5\|GB51383.1PA | CYP6AS19 |
| Apis_mellifera | tr\|A0A088AC40\|A0A088AC40_APIME | CYP6BC1 |
| Apis_mellifera | tr\|A0A088A7G1\|A0A088A7G1_APIME | CYP6BD1 |
| Apis_mellifera | tr\|A0A088A673\|A0A088A673_APIME | CYP6BE1 |
| Apis_mellifera | tr\|A0A087ZXU2\|A0A087ZXU2_APIME | CYP9P1 |
| Apis_mellifera | tr\|A0A087ZXU3\|A0A087ZXU3_APIME | CYP9P2 |
| Apis_mellifera | tr\|A0A087ZXU5\|A0A087ZXU5_APIME | CYP9Q1 |
| Apis_mellifera | tr\|A0A087ZXV8\|A0A087ZXV8_APIME | CYP9Q2 |
| Apis_mellifera | tr\|A0A087ZXV9\|A0A087ZXV9_APIME | CYP9Q3 |
| Apis_mellifera | tr\|A0A087ZXU8\|A0A087ZXU8_APIME | CYP9R1 |
| Apis_mellifera | tr\|A0A087ZXU7\|A0A087ZXU7_APIME | CYP9S1 |
| Apis_mellifera | tr\|A0A088A9W1\|A0A088A9W1_APIME | CYP15A1 |
| Apis_mellifera | tr\|A0A088ASU9\|A0A088ASU9_APIME | CYP18A1 |
| Apis_mellifera | gnl\|Amel_4.5\|GB46015.1PA | CYP301A1 |
| Apis_mellifera | tr\|A0A088A457\|A0A088A457_APIME | CYP301B1 |
| Apis_mellifera | tr\|A0A088A954\|A0A088A954_APIME | CYP302A1 |
| Apis_mellifera | tr\|A0A088A8Q7\|A0A088A8Q7_APIME | CYP303A1 |
| Apis_mellifera | tr\|A0A088A9P7\|A0A088A9P7_APIME | CYP305D1 |
| Apis_mellifera | gnl\|Amel_4.5\|GB54743.1PA | CYP306A1 |
| Apis_mellifera | tr\|A0A088AR99\|A0A088AR99_APIME | CYP307B1 |
| Apis_mellifera | tr\|Q306I2\|Q306I2_APIME | CYP314A1 |
| Apis_mellifera | gnl\|Amel_4.5\|GB53709.1PA | CYP315A1 |
| Apis_mellifera | tr\|A0A087ZVL6\|A0A087ZVL6_APIME | CYP334A1 |
| Apis_mellifera | tr\|A0A088AVB4\|A0A088AVB4_APIME | CYP336A1 |
| Apis_mellifera | tr\|A0A087ZXS6\|A0A087ZXS6_APIME | CYP343A1 |
| Apis_mellifera | tr\|A0A088A938\|A0A088A938_APIME | CYP369A1 |
| Apis_florea | XP_012350780.1 | CYP4G11_ortholog |
| Apis_florea | XP_003695320.2 | CYP4AA1_ortholog |
| Apis_florea | XP_012348791.1 | CYP4AB3 |
| Apis_florea | XP_003697419.1 | CYP6AQ1_ortholog |
| Apis_florea | XP_003694553.1 | CYP6AS2_ortholog |
| Apis_florea | XP_003694561.1 | CYP6AS3_ortholog |
| Apis_florea | XP_003694562.1 | CYP6AS4_ortholog |
| Apis_florea | XP_003694559.2 | CYP6AS5_ortholog |
| Apis_florea | XP_012343195.1 | CYP6AS7_ortholog |
| Apis_florea | XP_003694542.2(B) | CYP6AS9_ortholog |
| Apis_florea | XP_003694542.2 | CYP6AS10_ortholog |
| Apis_florea | XP_012343088.1(B) | CYP6AS11 |
| Apis_florea | XP_012343142.1 | CYP6AS12_ortholog |
| Apis_florea | XP_012343088.1 | CYP6AS14_ortholog |
| Apis_florea | XP_003698349.1 | CYP6AS15_ortholog |
| Apis_florea | XP_003694554.1 | CYP6AS16_ortholog |
| Apis_florea | XP_003696804.1 | CYP6AS19_ortholog |
| Apis_florea | XP_003690779.1 | CYP6BC1_ortholog |
| Apis_florea | XP_012348817.1 | CYP6BD1_ortholog |
| Apis_florea | XP_012346782.1 | CYP6BE1_ortholog |
| Apis_florea | XP_003697814.1 | CYP9Q1_ortholog |
| Apis_florea | XP_003697813.2(B) | CYP9R1_ortholog |
| Apis_florea | XP_003697813.2 | CYP9S1_ortholog |
| Apis_florea | XP_012341160.1 | CYP15A1_ortholog |
| Apis_florea | XP_012343956.1 | CYP18A1_ortholog |
| Apis_florea | XP_012342166.1 | CYP301A1_ortholog |
| Apis_florea | XP_012342168.1 | CYP301B1_ortholog |
| Apis_florea | XP_012342090.1 | CYP303A1 |
| Apis_florea | XP_003693153.1 | CYP305D1_ortholog_Apis |
| Apis_florea | XP_003696746.1 | CYP314A1_ortholog |
| Apis_florea | XP_012349282.1 | CYP315A1_ortholog |
| Apis_florea | XP_003690447.2 | CYP334A1_ortholog |
| Apis_florea | XP_003697031.1 | CYP336A1_ortholog |
| Apis_florea | XP_012346798.1 | CYP343A1_ortholog |
| Apis_florea | XP_012339807.1 | CYP369A1_ortholog |
| Bombus_terrestris | XP_003399611.1 | CYP4G11 |
| Bombus_terrestris | XP_003396921.1 | CYP4AA1 |
| Bombus_terrestris | XP_003398964.1 | CYP4AB3 |
| Bombus_terrestris | XP_003401971.1 | CYP4AV1 |
| Bombus_terrestris | XP_012170233.1 | CYP6AQ26 |
| Bombus_terrestris | XP_020721551.1 | CYP6AQ27 |
| Bombus_terrestris | XP_003399696.2 | CYP6AQ28 |
| Bombus_terrestris | XP_012170225.1 | CYP6AQ29 |
| Bombus_terrestris | XP_012170221.2 | CYP6AQ31 |
| Bombus_terrestris | XP_003393848.1 | CYP6AQ33 |
| Bombus_terrestris | XP_020721963.1(B) | CYP6AS5 |
| Bombus_terrestris | XP_003400119.1 | CYP6AS7 |
| Bombus_terrestris | XP_012170861.1 | CYP6AS10 |
| Bombus_terrestris | XP_020721963.1 | CYP6AS12 |
| Bombus_terrestris | XP_003400131.1 | CYP6AS13 |
| Bombus_terrestris | XP_003395878.1 | CYP6AS19 |
| Bombus_terrestris | XP_012170857.1 | CYP6AS72 |
| Bombus_terrestris | XP_012170859.1 | CYP6AS73 |
| Bombus_terrestris | XP_003400129.2(B) | CYP6AS74 |
| Bombus_terrestris | XP_003400129.2 | CYP6AS75 |
| Bombus_terrestris | XP_003400126.1 | CYP6AS76 |
| Bombus_terrestris | XP_012170855.1 | CYP6AS77 |
| Bombus_terrestris | XP_012168947.1 | CYP6BC1 |
| Bombus_terrestris | XP_012176083.1 | CYP6BD1 |
| Bombus_terrestris | XP_003398646.1 | CYP6BE1 |
| Bombus_terrestris | XM_020862886.1 | CYP9P1 |
| Bombus_terrestris | XP_003393388.3 | CYP9P2 |
| Bombus_terrestris | XP_003393377.1 | CYP9Q4 |
| Bombus_terrestris | XP_003393376.1 | CYP9Q5 |
| Bombus_terrestris | XM_003403348.2 | CYP9Q6 |
| Bombus_terrestris | XP_003393379.1 | CYP9R1 |
| Bombus_terrestris | XP_020723850.1 | CYP9S1 |
| Bombus_terrestris | XP_003396707.1 | CYP15A1 |
| Bombus_terrestris | XP_003400174.1 | CYP18A1 |
| Bombus_terrestris | XP_003396087.1 | CYP301A1 |
| Bombus_terrestris | XP_012164770.1 | CYP301B1 |
| Bombus_terrestris | XP_020718317.1 | CYP302A1 |
| Bombus_terrestris | XP_003397002.1 | CYP303A1 |
| Bombus_terrestris | XP_003396668.1 | CYP305D1 |
| Bombus_terrestris | XP_003400316.2 | CYP306A1 |
| Bombus_terrestris | XP_003400661.1 | CYP307B1 |
| Bombus_terrestris | XP_012164110.1 | CYP314A1 |
| Bombus_terrestris | XP_003394612.1 | CYP315A1 |
| Bombus_terrestris | XP_003397788.2 | CYP334A1 |
| Bombus_terrestris | XP_020723943.1 | CYP336A22 |
| Bombus_terrestris | XP_003393865.1 | CYP336A23 |
| Bombus_terrestris | XP_003393867.1 | CYP336A24 |
| Bombus_terrestris | XP_003393866.1 | CYP336A25 |
| Bombus_terrestris | XP_003397349.1 | CYP369A1 |
| Bombus_impatiens | XP_003491030.1 | CYP4G11 |
| Bombus_impatiens | XP_003492571.1 | CYP4AA1 |
| Bombus_impatiens | XP_003485532.1 | CYP4AB3 |
| Bombus_impatiens | XP_012246875.1 | CYP4AV1 |
| Bombus_impatiens | XP_003491921.1 | CYP6AQ26 |
| Bombus_impatiens | XP_003491956.1 | CYP6AQ29 |
| Bombus_impatiens | XP_012244660.1 | CYP6AQ42 |
| Bombus_impatiens | XP_012244655.1 | CYP6AQ43 |
| Bombus_impatiens | XP_012244656.1 | CYP6AQ44 |
| Bombus_impatiens | XP_012244658.1 | CYP6AQ31 |
| Bombus_impatiens | XP_003494723.1 | CYP6AQ33 |
| Bombus_impatiens | XP_012244661.1 | CYP6AQ45 |
| Bombus_impatiens | XP_003493216.1 | CYP6AS5 |
| Bombus_impatiens | XP_003493239.1 | CYP6AS7 |
| Bombus_impatiens | XP_003494397.2 | CYP6AS10 |
| Bombus_impatiens | XP_003493234.1 | CYP6AS12 |
| Bombus_impatiens | XP_003494516.1 | CYP6AS13 |
| Bombus_impatiens | XP_003487611.1 | CYP6AS19 |
| Bombus_impatiens | XP_003494397.2(B) | CYP6AS72 |
| Bombus_impatiens | XP_003494524.1 | CYP6AS73 |
| Bombus_impatiens | XP_012248456.1 | CYP6AS74 |
| Bombus_impatiens | XP_012248456.1(B) | CYP6AS75 |
| Bombus_impatiens | XP_003494525.1 | CYP6AS76 |
| Bombus_impatiens | XP_012246661.1 | CYP6AS77 |
| Bombus_impatiens | XP_012237477.1 | CYP6BC1 |
| Bombus_impatiens | XP_012236281.1 | CYP6BD1 |
| Bombus_impatiens | XP_012240213.1 | CYP6BE1 |
| Bombus_impatiens | XP_012250020.1 | CYP9P1 |
| Bombus_impatiens | XP_003486066.2 | CYP9Q4 |
| Bombus_impatiens | XP_003486050.1 | CYP9Q5 |
| Bombus_impatiens | XP_003486066.2(B) | CYP9Q6 |
| Bombus_impatiens | XP_012250003.1 | CYP9R1 |
| Bombus_impatiens | XP_003484728.1 | CYP15A1 |
| Bombus_impatiens | XP_003494528.1 | CYP18A1 |
| Bombus_impatiens | XP_003484880.1 | CYP301A1 |
| Bombus_impatiens | XP_003484923.1 | CYP301B1 |
| Bombus_impatiens | XP_012245116.1 | CYP302A1 |
| Bombus_impatiens | XP_003488458.1 | CYP303A1 |
| Bombus_impatiens | XP_003484727.1 | CYP305D1 |
| Bombus_impatiens | XP_012248622.1 | CYP306A1 |
| Bombus_impatiens | XP_003485019.1 | CYP307B1 |
| Bombus_impatiens | XP_012243763.1 | CYP314A1 |
| Bombus_impatiens | XP_003485328.2 | CYP315A1 |
| Bombus_impatiens | XP_003487290.2 | CYP334A1 |
| Bombus_impatiens | XP_012236162.1 | CYP336A23 |
| Bombus_impatiens | XP_003484581.1 | CYP336A24 |
| Bombus_impatiens | XP_003493197.1 | CYP369A1 |
| Megachile_rotundata | XP_012145465.1 | CYP4G11 |
| Megachile_rotundata | XP_003700755.1 | CYP4G202 |
| Megachile_rotundata | XP_012137854.1 | CYP4AA1 |
| Megachile_rotundata | XP_003699552.2 | CYP4AV1 |
| Megachile_rotundata | XP_012136078.1 | CYP6AQ52 |
| Megachile_rotundata | XP_012136079.1 | CYP6AQ53 |
| Megachile_rotundata | XP_003701310.1 | CYP6AQ54 |
| Megachile_rotundata | XP_012146067.1 | CYP6AS108 |
| Megachile_rotundata | XP_012150537.1 | CYP6AS109 |
| Megachile_rotundata | XP_012136792.1 | CYP6AS110 |
| Megachile_rotundata | XP_012143066.1 | CYP6AS111 |
| Megachile_rotundata | XP_003704416.1 | CYP6AS112 |
| Megachile_rotundata | XP_012143067.1 | CYP6AS113 |
| Megachile_rotundata | XP_012143073.1 | CYP6AS114 |
| Megachile_rotundata | XP_012143072.1 | CYP6AS115 |
| Megachile_rotundata | XP_003704412.1 | CYP6AS116 |
| Megachile_rotundata | XP_012143062.1 | CYP6AS117 |
| Megachile_rotundata | XP_003704278.1 | CYP6AS118 |
| Megachile_rotundata | XP_012140372.1 | CYP6AS119 |
| Megachile_rotundata | XP_003704414.1 | CYP6AS120 |
| Megachile_rotundata | XP_003703200.1 | CYP6BC1 |
| Megachile_rotundata | XP_003699913.1 | CYP6BD1 |
| Megachile_rotundata | XP_012153915.1 | CYP6BE1 |
| Megachile_rotundata | XP_012153916.1 | CYP6BE1P |
| Megachile_rotundata | XP_003703411.1 | CYP9DN1 |
| Megachile_rotundata | XP_012145771.1 | CYP9P2 |
| Megachile_rotundata | XP_012145774.1 | CYP9P22 |
| Megachile_rotundata | XP_012145773.1 | CYP9P23 |
| Megachile_rotundata | XP_003705491.1 | CYP9R1 |
| Megachile_rotundata | XP_003705489.1 | CYP9R58 |
| Megachile_rotundata | XP_012145777.1 | CYP9R59 |
| Megachile_rotundata | XP_003705488.1 | CYP9DM1 |
| Megachile_rotundata | XP_003705490.2 | CYP9DM2 |
| Megachile_rotundata | XP_012138027.1 | CYP15A1 |
| Megachile_rotundata | XP_012142657.1 | CYP18A1 |
| Megachile_rotundata | XP_003703366.2 | CYP301A1 |
| Megachile_rotundata | XP_003703365.1 | CYP301B1 |
| Megachile_rotundata | XP_012135500.1 | CYP302A1 |
| Megachile_rotundata | XP_012153072.1 | CYP303A1 |
| Megachile_rotundata | XP_003702175.1 | CYP305D1 |
| Megachile_rotundata | XP_012139366.1 | CYP307B1 |
| Megachile_rotundata | XP_012150013.1 | CYP314A1 |
| Megachile_rotundata | XP_003704053.1 | CYP315A1 |
| Megachile_rotundata | XP_003700083.1 | CYP334A1 |
| Megachile_rotundata | XP_003702293.1 | CYP336A33 |
| Megachile_rotundata | XP_003702219.1 | CYP336A34 |
| Megachile_rotundata | XP_012138246.1 | CYP336M1 |
| Megachile_rotundata | XP_012138467.1 | CYP343A1 |
| Megachile_rotundata | XP_003703633.1 | CYP369A1 |
