# Supplementary material for: Genomic insights into neonicotinoid sensitivity in the solitary bee Osmia bicornis
Source: PLoS Genet. 2019 Feb 4;15(2):e1007903. doi: 10.1371/journal.pgen.1007903 (PMC6375640; doi:10.1371/journal.pgen.1007903)
Supplement: S6 Table — (DOCX) [file pgen.1007903.s012.docx]

| Strain | Insecticide | LC50-value [mg/L^-1^] | 95% limits | Slope (+/- SE) | RR |
| --- | --- | --- | --- | --- | --- |
| Control | Thiacloprid | 17.03 | 8.4-27.9 | 1.2 +/- 0.14 | 1 |
| CYP9BU1  CYP9BU2 | Thiacloprid  Thiacloprid | 65.7  17.5 | 31.2-111.4  4.9-37.2 | - 1. +/- 0.12   1.2 +/- 0.13 | 3.8  1.1 |
| Control | Imidacloprid | 165.03 | 131-207.1 | 1.7 +/- 0.12 | 1 |
| CYP9BU1 | Imidacloprid | 85.9 | 61.9-115.2 | 1.3 +/- 0.13 | 0.5 |
| CYP9BU2 | Imidacloprid | 135.4 | 97.2-185.5 | 1.5 +/- 0.14 | 0.8 |
