# Supplementary material for: Genomic insights into neonicotinoid sensitivity in the solitary bee Osmia bicornis
Source: PLoS Genet. 2019 Feb 4;15(2):e1007903. doi: 10.1371/journal.pgen.1007903 (PMC6375640; doi:10.1371/journal.pgen.1007903)
Supplement: S7 Table — (DOCX) [file pgen.1007903.s013.docx]

| **Gene ID** | **log2(fold_change)** | **Fold_change** | **q_value** | **Blast_annotation** |
| --- | --- | --- | --- | --- |
| g29168 | 2.61343 | 6.12 | 0.0115 | predicted protein, partial [Nematostella vectensis] |
| g32741 | 1.90193 | 3.74 | 0.0115 | uncharacterized protein LOC100880045 isoform X1 [Megachile rotundata] |
| g31521 | 1.81208 | 3.51 | 0.0115 | uncharacterized protein LOC105664198 [Megachile rotundata] |
| g30471 | 1.63723 | 3.11 | 0.0115 | protein lethal(2)essential for life-like [Megachile rotundata] |
| g4165 | 1.58584 | 3.00 | 0.0115 | venom allergen 3-like [Megachile rotundata] |
| g914 | 1.33731 | 2.53 | 0.0115 | uncharacterized protein LOC100864565 [Apis florea] |
| g32438 | 1.32947 | 2.51 | 0.0115 | hypothetical protein WH47_07919 [Habropoda laboriosa] |
| g2479 | 1.32186 | 2.50 | 0.0288 | uncharacterized protein LOC100876166 [Megachile rotundata] |
| g29351 | 1.31515 | 2.49 | 0.0115 | L-lactate dehydrogenase-like [Megachile rotundata] |
| g6029 | 1.2055 | 2.31 | 0.0115 | chymotrypsin inhibitor-like [Megachile rotundata] |
| g2245 | 1.19091 | 2.28 | 0.0288 | uncharacterized protein LOC100877442 [Megachile rotundata] |
| g30763 | 1.16652 | 2.24 | 0.0115 | pancreatic triacylglycerol lipase-like [Megachile rotundata] |
| g29252 | 1.12796 | 2.19 | 0.0115 | probable G-protein coupled receptor No9 [Megachile rotundata] |
| g6850 | 1.0508 | 2.07 | 0.0115 | heat shock 70 kDa protein cognate 4 isoform X1 [Megachile rotundata] |
| g1945 | 1.01164 | 2.02 | 0.0115 | putative helicase mov-10-B.1 [Megachile rotundata] |
| g5989 | 0.984621 | 1.98 | 0.0115 | 15-hydroxyprostaglandin dehydrogenase [NAD(+)]-like isoform X1 [Megachile rotundata] |
| g8774 | 0.846198 | 1.80 | 0.0115 | uncharacterized protein LOC105662115 isoform X1 [Megachile rotundata] |
| g2055 | 0.817423 | 1.76 | 0.0115 | Terminal uridylyltransferase 7 [Dufourea novaeangliae] |
| g4205 | 0.763362 | 1.70 | 0.0115 | adenosine deaminase CECR1-like [Megachile rotundata] |
| g2478 | 0.732056 | 1.66 | 0.0205 | uncharacterized protein LOC100876166 [Megachile rotundata] |
| g4392 | 0.731108 | 1.66 | 0.0115 | cytochrome b5-related protein-like [Megachile rotundata] |
| g8757 | 0.730712 | 1.66 | 0.0115 | uncharacterized protein LOC100883975 isoform X3 [Megachile rotundata] |
| g5892 | 0.706928 | 1.63 | 0.0115 | FK506-binding protein 59 isoform X1 [Megachile rotundata] |
| g510 | 0.684204 | 1.61 | 0.0115 | probable salivary secreted peptide [Megachile rotundata] |
| g6678 | 0.683142 | 1.61 | 0.0421 | leucine-rich repeat-containing G-protein coupled receptor 5 [Megachile rotundata] |
| g8744 | 0.660821 | 1.58 | 0.0115 | trypsin-1-like [Apis florea] |
| g9698 | 0.654039 | 1.57 | 0.0421 | transferrin [Megachile rotundata] |
| g3510 | -0.55036 | 0.68284782 | 0.028803 | histone acetyltransferase p300 isoform X6 [Megachile rotundata] |
| g6134 | -0.55833 | 0.67908685 | 0.047852 | patatin-like phospholipase domain-containing protein 2 isoform X3 [Megachile rotundata] |
| g2209 | -0.57773 | 0.67001951 | 0.028803 | PHD finger protein rhinoceros [Megachile rotundata] |
| g30480 | -0.5983 | 0.66053321 | 0.042105 | SH3 and multiple ankyrin repeat domains protein 1 isoform X2 [Bombus terrestris] |
| g31841 | -0.60485 | 0.65754065 | 0.020508 | uncharacterized protein LOC100874903 [Megachile rotundata] |
| g3023 | -0.63261 | 0.64500981 | 0.020508 | adenomatous polyposis coli homolog isoform X1 [Megachile rotundata] |
| g32355 | -0.64541 | 0.63931329 | 0.035467 | open rectifier potassium channel protein 1 isoform X2 [Megachile rotundata] |
| g4337 | -0.65373 | 0.63563567 | 0.035467 | tyrosine 3-monooxygenase [Megachile rotundata] |
| g33429 | -0.65418 | 0.63543612 | 0.035467 | forkhead box protein P1 isoform X4 [Bombus terrestris] |
| g5490 | -0.65862 | 0.63348616 | 0.011507 | mucin-19-like isoform X2 [Megachile rotundata] |
| g33394 | -0.69961 | 0.61573949 | 0.011507 | calmodulin-binding transcription activator 2 isoform X3 [Megachile rotundata] |
| g5239 | -0.71154 | 0.61066878 | 0.047852 | uncharacterized protein LOC100876062 isoform X3 [Megachile rotundata] |
| g4499 | -0.72643 | 0.60439851 | 0.011507 | Mitochondrial sodium/hydrogen exchanger 9B2 [Habropoda laboriosa] |
| g2457 | -0.74237 | 0.59775492 | 0.011507 | Flightin [Melipona quadrifasciata] |
| g3434 | -0.7529 | 0.59340871 | 0.047852 | LOW QUALITY PROTEIN: protein turtle [Megachile rotundata] |
| g33003 | -0.76357 | 0.58903612 | 0.035467 | nephrin-like [Megachile rotundata] |
| g9051 | -0.77122 | 0.58592341 | 0.011507 | diacylglycerol kinase theta-like isoform X6 [Apis mellifera] |
| g1761 | -0.78087 | 0.58201571 | 0.011507 | uncharacterized protein LOC100883603 [Megachile rotundata] |
| g31063 | -0.80071 | 0.57406659 | 0.011507 | protein unc-80 homolog [Megachile rotundata] |
| g2032 | -0.80449 | 0.57256643 | 0.011507 | uncharacterized protein R02F2.2 isoform X4 [Megachile rotundata] |
| g5381 | -0.80484 | 0.57242715 | 0.011507 | solute carrier family 12 member 6-like isoform X5 [Apis dorsata] |
| g727 | -0.84391 | 0.55713119 | 0.011507 | microtubule-associated protein futsch, partial [Megachile rotundata] |
| g29400 | -0.85855 | 0.55150849 | 0.011507 | glutamate receptor ionotropic, NMDA 2B [Megachile rotundata] |
| g6887 | -0.86059 | 0.55072614 | 0.011507 | high-affinity choline transporter 1 [Megachile rotundata] |
| g8844 | -0.86142 | 0.55041168 | 0.011507 | putative uncharacterized protein DDB_G0271606 isoform X4 [Apis florea] |
| g1191 | -0.87234 | 0.54625974 | 0.011507 | actin, muscle [Nasonia vitripennis] |
| g5622 | -0.8846 | 0.54163843 | 0.020508 | acetylcholinesterase [Dufourea novaeangliae] |
| g4717 | -0.89526 | 0.53765141 | 0.035467 | zinc finger CCCH domain-containing protein 28-like [Bombus impatiens] |
| g32954 | -0.91842 | 0.52908668 | 0.011507 | fibrillin-2-like isoform X1 [Megachile rotundata] |
| g2347 | -0.95488 | 0.51588521 | 0.011507 | ras-specific guanine nucleotide-releasing factor 1-like [Megachile rotundata] |
| g2193 | -0.95671 | 0.51522911 | 0.028803 | heterogeneous nuclear ribonucleoprotein L isoform X1 [Apis mellifera] |
| g3706 | -0.98556 | 0.5050307 | 0.011507 | sodium channel protein 60E isoform X1 [Bombus impatiens] |
| g8971 | -0.99859 | 0.50049064 | 0.011507 | tachykinins isoform X1 [Megachile rotundata] |
| g6940 | -1.00445 | 0.49846012 | 0.011507 | homeobox protein six1b [Megachile rotundata] |
| g774 | -1.00552 | 0.49809057 | 0.011507 | 4-nitrophenylphosphatase-like isoform X1 [Megachile rotundata] |
| g1098 | -1.04733 | 0.48386282 | 0.011507 | protein unc-13 homolog B [Megachile rotundata] |
| g9923 | -1.0626 | 0.47876845 | 0.011507 | protein unc-79 homolog [Megachile rotundata] |
| g6887 | -1.08006 | 0.47300915 | 0.042105 | high-affinity choline transporter 1 [Megachile rotundata] |
| g8960 | -1.08504 | 0.4713792 | 0.011507 | GTPase-activating Rap/Ran-GAP domain-like protein 3 isoform X3 [Bombus impatiens] |
| g29296 | -1.21154 | 0.43180744 | 0.011507 | potassium channel subfamily T member 2 [Megachile rotundata] |
| g23031 | -1.24033 | 0.42327583 | 0.011507 | nose resistant to fluoxetine protein 6-like isoform X1 [Megachile rotundata] |
| g34176 | -1.34165 | 0.39456913 | 0.011507 | purine nucleoside phosphorylase-like isoform X1 [Megachile rotundata] |
| g15329 | -1.34956 | 0.39241171 | 0.011507 | elongation of very long chain fatty acids protein 1-like [Dufourea novaeangliae] |
| g1386 | -1.3943 | 0.38042923 | 0.035467 | 1-phosphatidylinositol 4,5-bisphosphate phosphodiesterase epsilon-1 [Apis mellifera] |
| g34176 | -1.40503 | 0.37761029 | 0.011507 | purine nucleoside phosphorylase-like isoform X1 [Megachile rotundata] |
| g12942 | -1.41069 | 0.37613175 | 0.011507 | cytochrome P450 6B1-like [Megachile rotundata] |
| g5456 | -1.53869 | 0.34419785 | 0.011507 | farnesyl pyrophosphate synthase isoform X3 [Megachile rotundata] |
| g23893 | -1.59919 | 0.33006224 | 0.011507 | LOW QUALITY PROTEIN: uncharacterized protein LOC105663869 [Megachile rotundata] |
| g8515 | -1.76211 | 0.29481667 | 0.011507 | PREDICTED: nose resistant to fluoxetine protein 6-like isoform X1 [Megachile rotundata] |
| g19074 | -1.92468 | 0.26339868 | 0.011507 | elongation of very long chain fatty acids protein 1-like [Dufourea novaeangliae] |
| g27717 | -2.14551 | 0.22601493 | 0.011507 | piggyBac transposable element-derived protein 4-like [Polistes dominula] |
| g5701 | -2.99155 | 0.12573428 | 0.011507 | 4-coumarate--CoA ligase 1-like [Megachile rotundata] |
