# Supplementary material for: Genomic insights into neonicotinoid sensitivity in the solitary bee Osmia bicornis
Source: PLoS Genet. 2019 Feb 4;15(2):e1007903. doi: 10.1371/journal.pgen.1007903 (PMC6375640; doi:10.1371/journal.pgen.1007903)
Supplement: S8 Table — (DOCX) [file pgen.1007903.s014.docx]

| **Gene ID** | **log2(fold_change)** | **Fold_change** | **q_value** | **Blast_annotation** |
| --- | --- | --- | --- | --- |
| g2479 | 3.54398 | 11.66 | 0.0115 | uncharacterized protein LOC100876166 [Megachile rotundata] |
| g2478 | 2.05938 | 4.17 | 0.0115 | uncharacterized protein LOC100876166 [Megachile rotundata] |
| g29168 | 2.01239 | 4.03 | 0.0115 | predicted protein, partial [Nematostella vectensis] |
| g32438 | 2.01098 | 4.03 | 0.0115 | hypothetical protein WH47_07919 [Habropoda laboriosa] |
| g4392 | 1.60423 | 3.04 | 0.0115 | cytochrome b5-related protein-like [Megachile rotundata] |
| g9698 | 1.58803 | 3.01 | 0.0115 | transferrin [Megachile rotundata] |
| g30471 | 1.23436 | 2.35 | 0.0115 | protein lethal(2)essential for life-like [Megachile rotundata] |
| g31064 | 1.1261 | 2.18 | 0.0115 | carboxypeptidase Q-like isoform X1 [Megachile rotundata] |
| g29351 | 1.09507 | 2.14 | 0.0115 | L-lactate dehydrogenase-like [Megachile rotundata] |
| g3206 | 0.990934 | 1.99 | 0.0115 | cytochrome b561 domain-containing protein 1-like [Megachile rotundata] |
| g33652 | 0.873628 | 1.83 | 0.0115 | uncharacterized protein LOC100881220 [Megachile rotundata] |
| g4165 | 0.744423 | 1.68 | 0.0115 | venom allergen 3-like [Megachile rotundata] |
| g6850 | 0.731932 | 1.66 | 0.0115 | heat shock 70 kDa protein cognate 4 isoform X1 [Megachile rotundata] |
| g914 | 0.718926 | 1.65 | 0.0205 | uncharacterized protein LOC100864565 [Apis florea] |
| g2055 | 0.639452 | 1.56 | 0.0115 | Terminal uridylyltransferase 7 [Dufourea novaeangliae] |
| g4745 | 0.611184 | 1.53 | 0.0479 | mucin-19-like [Megachile rotundata] |
| g31611 | -0.58712 | 0.665671 | 0.047852 | carboxypeptidase B-like [Megachile rotundata] |
| g1761 | -0.63871 | 0.642287 | 0.047852 | uncharacterized protein LOC100883603 [Megachile rotundata] |
| g1888 | -0.662 | 0.632004 | 0.035467 | UDP-glucuronosyltransferase 1-2-like [Megachile rotundata] |
| g1430 | -0.69267 | 0.618708 | 0.042105 | phenoloxidase 2 [Megachile rotundata] |
| g32954 | -0.69531 | 0.617576 | 0.042105 | fibrillin-2-like isoform X1 [Megachile rotundata] |
| g31841 | -0.82441 | 0.564713 | 0.011507 | uncharacterized protein LOC100874903 [Megachile rotundata] |
| g5456 | -0.83398 | 0.560978 | 0.020508 | farnesyl pyrophosphate synthase isoform X3 [Megachile rotundata] |
| g34065 | -0.84947 | 0.554987 | 0.011507 | sodium-dependent nutrient amino acid transporter 1-like [Megachile rotundata] |
| g2078 | -0.85115 | 0.554343 | 0.047852 | Protein kinase DC2 [Habropoda laboriosa] |
| g5009 | -0.8699 | 0.547184 | 0.011507 | uncharacterized protein LOC100880123 [Megachile rotundata] |
| g6999 | -0.88104 | 0.542975 | 0.011507 | uncharacterized protein LOC100876107 [Megachile rotundata] |
| g23031 | -0.94725 | 0.518619 | 0.011507 | nose resistant to fluoxetine protein 6-like isoform X1 [Megachile rotundata] |
| g27717 | -1.08384 | 0.471771 | 0.035467 | piggyBac transposable element-derived protein 4-like [Polistes dominula] |
| g8515 | -1.10787 | 0.463979 | 0.011507 | nose resistant to fluoxetine protein 6-like isoform X1 [Megachile rotundata] |
| g15329 | -1.14036 | 0.453646 | 0.011507 | elongation of very long chain fatty acids protein 1-like [Dufourea novaeangliae] |
| g33322 | -1.1853 | 0.439733 | 0.020508 | facilitated trehalose transporter Tret1-like isoform X2 [Megachile rotundata] |
| g5563 | -1.34732 | 0.393021 | 0.011507 | serine protease nudel isoform X2 [Megachile rotundata] |
| g33840 | -1.71225 | 0.305184 | 0.011507 | ornithine decarboxylase 2-like isoform X1 [Megachile rotundata] |
