# Supplementary material for: Genomic insights into neonicotinoid sensitivity in the solitary bee Osmia bicornis
Source: PLoS Genet. 2019 Feb 4;15(2):e1007903. doi: 10.1371/journal.pgen.1007903 (PMC6375640; doi:10.1371/journal.pgen.1007903)
Supplement: S9 Table — (DOCX) [file pgen.1007903.s015.docx]

| Model | BIC.Fig1 | BIC.FigS2 | BIC.FigS3 |
| --- | --- | --- | --- |
| Blosum62 | 289596 | 92690.98 | 88758.11 |
| Blosum62+G | 284368.4 | 91658.37 | 86932.04 |
| Blosum62+G+I | 284368.4 | 91613.16 | 86913.09 |
| Blosum62+I | 289596 | 92551.45 | 88544.5 |
| cpREV | 287041.5 | 92750.24 | 87865.02 |
| cpREV+G | 281741.1 | 91492.96 | 85923.94 |
| cpREV+G+I | 281741.1 | 91441.1 | 85893.33 |
| cpREV+I | 287041.5 | 92587.19 | 87630.13 |
| Dayhoff | 292947.6 | 94494.58 | 89241.59 |
| Dayhoff_DCMut | 292929 | 94485.82 | 89241.94 |
| Dayhoff_DCMut+G | 287022.6 | 92914.48 | 87211.7 |
| Dayhoff_DCMut+G+I | 287029.7 | 92894.59 | 87192.87 |
| Dayhoff_DCMut+I | 292818.1 | 94333.4 | 89020.67 |
| Dayhoff+G | 287028.3 | 92916.65 | 87206.92 |
| Dayhoff+G+I | 287035.4 | 92896.88 | 87188.09 |
| Dayhoff+I | 292833.7 | 94341.36 | 89019.95 |
| FLU | 293070.1 | 94707.23 | 89232.66 |
| FLU+G | 286235.8 | 92977.19 | 86776.44 |
| FLU+G+I | 286214.4 | 92569.81 | 86471.38 |
| FLU+I | 292574.2 | 94090.72 | 88718.05 |
| HIVb | 293058.7 | 94588.61 | 89266.81 |
| HIVb+G | 285941.6 | 92792.67 | 86645.18 |
| HIVb+G+I | 285941.6 | 92758.24 | 86606.32 |
| HIVb+I | 293058.7 | 94432.08 | 88963.49 |
| HIVw | 301765.4 | 98052.93 | 91932.07 |
| HIVw+G | 294301.6 | 96057.2 | 89269.02 |
| HIVw+G+I | 294301.6 | 96012.64 | 89215.94 |
| HIVw+I | 301765.4 | 97863.42 | 91574.98 |
| JTT | 287181.5 | 93038.12 | 87344.09 |
| JTT_DCMut | 287222.4 | 93045.1 | 87356.5 |
| JTT_DCMut+G | 281188.5 | 91563.09 | 85350.87 |
| JTT_DCMut+G+I | 281188.5 | 91522.84 | 85321.71 |
| JTT_DCMut+I | 287222.4 | 92890.46 | 87109.55 |
| JTT+G | 281124.6 | 91544.48 | 85330.95 |
| JTT+G+I | 281124.6 | 91503.97 | 85303.05 |
| JTT+I | 287181.5 | 92881.5 | 87099.01 |
| LG | 287464 | 92380.6 | 87469.79 |
| LG+G | 281231.2 | 90873.93 | 85359.39 |
| LG+G+I | 281231.2 | 90828.99 | 85337.45 |
| LG+I | 287464 | 92211.68 | 87233.66 |
| mtArt | 307941.6 | 98237.85 | 94342.64 |
| mtArt+G | 296572.3 | 95209.4 | 90011.03 |
| mtArt+G+I | 296572.3 | 95180.34 | 90010.11 |
| mtArt+I | 307941.6 | 98052.82 | 94235.19 |
| mtmam | 315790.9 | 101283.31 | 95337.79 |
| mtmam+G | 301463 | 97464.4 | 91150.2 |
| mtmam+G+I | 300404.9 | 97272.72 | 91012.68 |
| mtmam+I | 311633.2 | 100199.38 | 94880.67 |
| mtREV24 | 299202.1 | 96532.51 | 91705.33 |
| mtREV24+G | 291459.2 | 94557.2 | 88940.9 |
| mtREV24+G+I | 291459.2 | 94551.39 | 88947.25 |
| mtREV24+I | 299202.1 | 96455.61 | 91636.67 |
| MtZoa | 299497.2 | 96060.42 | 91358.78 |
| MtZoa+G | 289792.5 | 93471.36 | 87853.36 |
| MtZoa+G+I | 289792.5 | 93440.74 | 87854.1 |
| MtZoa+I | 299497.2 | 95875.99 | 91255.39 |
| RtREV | 291486.2 | 93416.07 | 89133.68 |
| RtREV+G | 285281.3 | 92004.04 | 86985.44 |
| RtREV+G+I | 285281.3 | 91952.91 | 86957.98 |
| RtREV+I | 291486.2 | 93241.44 | 88878.39 |
| VT | 292692.7 | 93949.71 | 89173.09 |
| VT+G | 286593.9 | 92432.62 | 87174.93 |
| VT+G+I | 286593.9 | 92406.12 | 87166.21 |
| VT+I | 292692.7 | 93840.75 | 89005.98 |
| WAG | 287519.1 | 92613.45 | 87786.92 |
| WAG+G | 282165 | 91425.73 | 85939.1 |
| WAG+G+I | 282165 | 91379.68 | 85909.42 |
| WAG+I | 287519.1 | 92462.44 | 87548.3 |
