# Supplementary material for: Genomic insights into neonicotinoid sensitivity in the solitary bee Osmia bicornis
Source: PLoS Genet. 2019 Feb 4;15(2):e1007903. doi: 10.1371/journal.pgen.1007903 (PMC6375640; doi:10.1371/journal.pgen.1007903)
Supplement: S10 Table — (DOCX) [file pgen.1007903.s016.docx]

| **Primer name** | **Sequence** | **Purpose** |
| --- | --- | --- |
| Ob_CYP9BU1_F1 | 5’-AACTGAGTCCGAAGAGCCGA-3’ | PCR validation (CYP9BU1) |
| Ob_CYP9BU1_R1 | 5’-CCT AGG CTC AAC TCG CAA CA-3’ | PCR validation (CYP9BU1) |
| Ob_CYP9BU2_F1 | 5’-GGGACCGGTGTTAGCAATGA-3’ | PCR validation (CYP9BU2) |
| Ob_CYP9BU2_R1 | 5’-AAGATCATTGGAACG CCTAGGT-3’ | PCR validation (CYP9BU2) |
| CYP9BU1_F | 5’-TCGAGATGGAGTACTTGACAATTACATTA-3’ | QPCR (CYP9BU1) |
| CYP9BU1_R | 5’-TATTGACTAACTCCATTAATCCAATCAACG-3’ | QPCR (CYP9BU1) |
| CYP9BU2_F | 5’-TATTGACTAACTCCATTAATCCAATCAACG-3’ | QPCR (CYP9BU2) |
| CYP9BU2_R | 5’-AATTCAAATTAACATTCTTCCTAGGTTCAAC-3’ | QPCR (CYP9BU2) |
| EF1_aF2 | ACGGACAAACTCGTGAGCAT | QPCR (reference gene) |
| EF1_aR2 | TCGTGTTTCAGAGTAGGGCG | QPCR (reference gene) |
| EF1_yF | CAAATGCTGGAACCTTGCCC | QPCR (reference gene) |
| EF1_yR | CCCGGAAAACTTCAGAGCCT | QPCR (reference gene) |
