# Supplementary material for: Genomic insights into neonicotinoid sensitivity in the solitary bee Osmia bicornis
Source: PLoS Genet. 2019 Feb 4;15(2):e1007903. doi: 10.1371/journal.pgen.1007903 (PMC6375640; doi:10.1371/journal.pgen.1007903)
Supplement: S11 Table — (DOCX) [file pgen.1007903.s017.docx]

| **Library** | **Library Type** | **Read Length** | **#Reads** | **Mate-pairs** | **Paired-end** | **Unknown** | **Single end** |
| --- | --- | --- | --- | --- | --- | --- | --- |
|  |  |  |  |  |  |  |  |
| LIB18336 | PE | 250 | 2.57E+08 | NA | 2.46E+08 | NA | 763028 |
| LIB20870 | MP | 250 | 21167258 | 9590982 | 7371608 | 1622480 | 3699374 |
| LIB20871 | MP | 250 | 25414620 | 11718560 | 8619532 | 1857624 | 4491321 |
| LIB20872 | MP | 250 | 60409146 | 27394388 | 20150396 | 3967026 | 10887036 |
| LIB20873 | MP | 250 | 56112894 | 25759634 | 18853132 | 3630924 | 10053251 |
| LIB20874 | MP | 250 | 65023054 | 30600378 | 21611308 | 3567408 | 11906057 |
| LIB20875 | MP | 250 | 63420654 | 29682108 | 22339088 | 3686004 | 11701585 |
