# Supplementary material for: Genomic insights into neonicotinoid sensitivity in the solitary bee Osmia bicornis
Source: PLoS Genet. 2019 Feb 4;15(2):e1007903. doi: 10.1371/journal.pgen.1007903 (PMC6375640; doi:10.1371/journal.pgen.1007903)
Supplement: S12 Table — (DOCX) [file pgen.1007903.s018.docx]

| **Library** | **Gel Fractions** | **ELF Library Size (kb)** | **Observed Insert Size** | **Std. Dev.** |
| --- | --- | --- | --- | --- |
| LIB20870 | ELF_Fraction3 | 14.1 | 1753.19 | 31890.74 |
| LIB20871 | ELF_Fraction4 | 12 | 2016.73 | 37076.56 |
| LIB20872 | ELF_Fraction5 | 10 | 2183.24 | 38646.75 |
| LIB20873 | ELF_Fraction6 | 8.2 | 2662.78 | 47468.03 |
| LIB20874 | ELF_Fraction7 | 6.7 | 2903.34 | 51844.91 |
| LIB20875 | ELF_Fraction8 | 5.3 | 2955.1 | 52491.61 |
