# Supplementary material for: Genomic insights into neonicotinoid sensitivity in the solitary bee Osmia bicornis
Source: PLoS Genet. 2019 Feb 4;15(2):e1007903. doi: 10.1371/journal.pgen.1007903 (PMC6375640; doi:10.1371/journal.pgen.1007903)
Supplement: S13 Table — (DOCX) [file pgen.1007903.s019.docx]

| **Library** | **Mean Insert size (bp)** | **#sequences (x 2 = paired sequences)** | **# nucleotides (bp)** | **%GC** | **Estimated Coverage** |
| --- | --- | --- | --- | --- | --- |
| LIB18336 | 650 | 257043048 | 64260762000 | 37.5 | 200.00 |
| LIB20870 | 1753 | 21167258 | 5291814500 | 43.9 | 5.26 |
| LIB20871 | 2017 | 25414620 | 6353655000 | 42.5 | 6.47 |
| LIB20872 | 2183 | 60409146 | 15102286500 | 42.4 | 15.00 |
| LIB20873 | 2663 | 56112894 | 14028223500 | 43.25 | 14.38 |
| LIB20874 | 2903 | 65023054 | 16255763500 | 42.66 | 17.45 |
| LIB20875 | 2955 | 63420654 | 15855163500 | 42.36 | 17.80 |
