# Supplementary material for: Genomic insights into neonicotinoid sensitivity in the solitary bee Osmia bicornis
Source: PLoS Genet. 2019 Feb 4;15(2):e1007903. doi: 10.1371/journal.pgen.1007903 (PMC6375640; doi:10.1371/journal.pgen.1007903)
Supplement: S14 Table — (DOCX) [file pgen.1007903.s020.docx]

| **Assembly pipeline steps** | **Number of Sequences** | **Total Length (bp)** | **N50 (bp) > 1000 bp** | **Ns** |
| --- | --- | --- | --- | --- |
| Discover-denovo (-v1) | 29194 | 235939786 | 303978 | 96400 |
| Redundans (-v2) | 10430 | 212904310 | 459933 | 13798 |
| L_RNA_Scaffolder + PEP_Scaffolder (-v3) | 10223 | 210598924 | 607766 | 33533 |
