# Supplementary material for: Genomic insights into neonicotinoid sensitivity in the solitary bee Osmia bicornis
Source: PLoS Genet. 2019 Feb 4;15(2):e1007903. doi: 10.1371/journal.pgen.1007903 (PMC6375640; doi:10.1371/journal.pgen.1007903)
Supplement: S15 Table — (DOCX) [file pgen.1007903.s021.docx]

| **Library** | **Mean Insert size** | **Reads mapped** | **%** | **Both in pair** | **%** | **Mean/Estimated Coverage** | **Mean MAPQ** |
| --- | --- | --- | --- | --- | --- | --- | --- |
| LIB18336 | 650 | 244,788,278 |  | 243,838,677 |  | 205.59 | 38.21 |
| LIB20870 | 1753 | 9,861,001 |  | 9,698,948 |  | 5.26 | 22.81 |
| LIB20871 | 2017 | 12,056,157 |  | 11,843,612 |  | 6.47 | 23.87 |
| LIB20872 | 2183 | 28,061,680 |  | 27,547,868 |  | 15.01 | 25.28 |
| LIB20873 | 2663 | 26,377,186 |  | 25,905,528 |  | 14.38 | 25.83 |
| LIB20874 | 2903 | 31,269,515 |  | 30,740,029 |  | 17.45 | 26.31 |
| LIB20875 | 2955 | 30,315,834 |  | 29,868,695 |  | 17.80 | 26.84 |
