# Supplementary material for: Genomic insights into neonicotinoid sensitivity in the solitary bee Osmia bicornis
Source: PLoS Genet. 2019 Feb 4;15(2):e1007903. doi: 10.1371/journal.pgen.1007903 (PMC6375640; doi:10.1371/journal.pgen.1007903)
Supplement: S16 Table — (DOCX) [file pgen.1007903.s022.docx]

| **Groups** | **Number of Proteins** | **Completeness** | **Total** | **Average** | **Percentage of Orthologs** |
| --- | --- | --- | --- | --- | --- |
| Complete | 244 | 98.39 | 277 | 1.14 | 11.89 |
| Group 1 | 65 | 98.48 | 69 | 1.06 | 6.15 |
| Group 2 | 53 | 94.64 | 59 | 1.11 | 11.32 |
| Group 3 | 61 | 100.00 | 71 | 1.16 | 13.11 |
| Group 4 | 65 | 100.00 | 78 | 1.20 | 16.92 |
| Partial | 246 | 99.19 | 292 | 1.19 | 15.04 |
| Group 1 | 66 | 100.00 | 72 | 1.09 | 9.09 |
| Group 2 | 54 | 96.43 | 62 | 1.15 | 14.81 |
| Group 3 | 61 | 100.00 | 75 | 1.23 | 16.39 |
| Group 4 | 65 | 100.00 | 83 | 1.23 | 20.00 |
