# Supplementary material for: Genomic insights into neonicotinoid sensitivity in the solitary bee Osmia bicornis
Source: PLoS Genet. 2019 Feb 4;15(2):e1007903. doi: 10.1371/journal.pgen.1007903 (PMC6375640; doi:10.1371/journal.pgen.1007903)
Supplement: S17 Table — (DOCX) [file pgen.1007903.s023.docx]

| Property | Number |
| --- | --- |
| Number of genes | 115480 |
| Number of genes in orthogroups | 101561 |
| Number of unassigned genes | 13919 |
| Percentage of genes in orthogroups | 87.9 |
| Percentage of unassigned genes | 12.1 |
| Number of orthogroups | 11184 |
| Number of species-specific orthogroups | 52 |
| Number of genes in species-specific orthogroups | 381 |
| Percentage of genes in species-specific orthogroups | 0.3 |
| Mean orthogroup size | 9.1 |
| Median orthogroup size | 7.0 |
| G50 (assigned genes) | 10 |
| G50 (all genes) | 9 |
| O50 (assigned genes) | 3113 |
| O50 (all genes) | 3847 |
| Number of orthogroups with all species present | 8134 |
| Number of single-copy orthogroups | 1767 |
