# Supplementary material for: Genomic insights into neonicotinoid sensitivity in the solitary bee Osmia bicornis
Source: PLoS Genet. 2019 Feb 4;15(2):e1007903. doi: 10.1371/journal.pgen.1007903 (PMC6375640; doi:10.1371/journal.pgen.1007903)
Supplement: S18 Table — AF: Apis florea, AM: Apis mellifera, BI: Bombus impatiens, BT: Bombus terrestris, MR: Megachile rotundata, OB: Osmia bicornis. (DOCX) [file pgen.1007903.s024.docx]

| **Property  (OG = orthogroups)** | **AF** | **AM** | **BI** | **BT** | **MR** | **OB** |
| --- | --- | --- | --- | --- | --- | --- |
| #genes | 17676 | 15314 | 15896 | 22091 | 26024 | 18479 |
| #genes in OG | 16845 | 11328 | 13364 | 21253 | 24228 | 14543 |
| #unassigned genes | 831 | 3986 | 2532 | 838 | 1796 | 3936 |
| %genes in OG | 95.3 | 74.0 | 84.1 | 96.2 | 93.1 | 78.7 |
| %unassigned genes | 4.7 | 26.0 | 15.9 | 3.8 | 6.9 | 21.3 |
| #OG containing species | 9874 | 10257 | 10352 | 9810 | 9743 | 9328 |
| %OG containing species | 88.3 | 91.7 | 92.6 | 87.7 | 87.1 | 83.4 |
| #species-specific OG | 3 | 4 | 4 | 6 | 16 | 19 |
| #genes in species-specific OG | 21 | 31 | 48 | 21 | 97 | 163 |
| %genes in species-specific OG | 0.1 | 0.2 | 0.3 | 0.1 | 0.4 | 0.9 |
