# Supplementary material for: Genomic insights into neonicotinoid sensitivity in the solitary bee Osmia bicornis
Source: PLoS Genet. 2019 Feb 4;15(2):e1007903. doi: 10.1371/journal.pgen.1007903 (PMC6375640; doi:10.1371/journal.pgen.1007903)
Supplement: S19 Table — (DOCX) [file pgen.1007903.s025.docx]

| **Stats based on ALL transcript contigs:** | **Stats based on ONLY LONGEST ISOFORM per 'GENE':** |
| --- | --- |
| Contig N10: 11189 | Contig N10: 9444 |
| Contig N20: 8173 | Contig N20: 6719 |
| Contig N30: 6424 | Contig N30: 4963 |
| Contig N40: 5134 | Contig N40: 3615 |
| Contig N50: 4002 | Contig N50: 2456 |
| Median contig length: 500 | Median contig length: 380 |
| Average contig: 1483.50 | Average contig: 962.41 |
| Total assembled bases: 137199608 | Total assembled bases: 62292057 |
