# Supplementary material for: Genomic insights into neonicotinoid sensitivity in the solitary bee Osmia bicornis
Source: PLoS Genet. 2019 Feb 4;15(2):e1007903. doi: 10.1371/journal.pgen.1007903 (PMC6375640; doi:10.1371/journal.pgen.1007903)
Supplement: S1 Text — (DOCX) [file pgen.1007903.s026.docx]

**Sequencing and pre-processing**

Genomic DNA extracted from a single male bee was used to prepare paired-end and mate-pair libraries with insert sizes of 450, 2k, 4.2k, 8.5k, 9.5k and 11.5k. Sequencing yielded more than half a billion reads (548,590,674) containing more than ~137 GB (137,147,668,500 bp) of raw data (S11 Table). All read files were quality checked using FastQC –v 0.10.1 [1] and those from small insert libraries pre-processed with trim_galore –v 0.4.4 (http://www.bioinformatics.babraham.ac.uk/projects/trim_galore/), while reads from mate-pair libraries were trimmed using NxTrim –v 0.4.1-0f17575 [2] with the –separate parameter. Insert-size estimation was performed by aligning all RF oriented reads against the –v1 assembly (see below) using bwa mem –v 0.7.12-r1039 [3] with default parameters. Read alignment statistics were generated using Picard’s CollectAlignmentSummaryMetrics tool –v 2.2.4 [4]. The observed insert size of all mate-pair libraries differed significantly from expected insert sizes (S12 Table). A maximum insert size of 4.5 KB was obtained, ranging from 500 – 4500 bp and this may have resulted from the low quantity of DNA used in library preparation in order to use DNA from a single male sample. Adapter trimmed reads were checked for possible contamination using FastqScreen –v 0.5.2 [5] with libraries from human (*Homo sapiens* GRCh38), mouse (*Mus musculus* GRCm38), *E. coli* (U00096.3), Enterobacteria phage phiX174 (NC_001422.1) and simulated bacterial and viral databases from DeconSeq –v 0.4.3 [6]. The vast majority of *O. bicornis* sequence reads did not map to these libraries, however, a small fraction of reads mapped multiple times to multiple genomes suggestive of repetitive sequences in these genomes.

**Genome size estimation using k-mers**

Genome size and characteristics (total and haploid genome length, percentage of repetitive content, and heterozygosity rate) were estimated from raw short read sequencing data via a statistical analysis of the k-mer profile using GenomeScope (<http://qb.cshl.edu/genomescope/>) [7]. The raw data displayed low genome heterozygosity (S1 Table), as expected from a single haploid DNA sample, with a uni modal (S4 Fig) k-mer distribution where the frequency of heterozygous k-mers displayed a single peak at 105X.

**Genome assembly**

Based on the statistics obtained from Genomescope, we chose DISCOVAR *de novo* –v 52488 [8] to assemble the PE Illumina data. Untrimmed raw sequences were fed to DISCOVAR *de novo* with default parameters to generate a first pass assembly (–v1 assembly). To scaffold the –v1 assembly, we further used Redundans –v 0.12a [9] with default parameters using all 7 (paired-end + mate-paired) libraries in ascending order along with their average insert sizes according to the estimated ELF fraction. All sequences smaller than 1000 bp were excluded from subsequent assembly steps. We iterated the Redundans run multiple times to utilize reads with different orientation as a result of PE contamination in Nextera mate-pair data. Using this iterative approach (using reads of all orientation: RF, FR and unknown orientation) the N50 of the resulting assembly (–v2 assembly) increased from 303 kb to 459 kb (S14 Table). A second scaffolding step was included to further scaffold the –v2 assembly using transcripts assembled from sex-specific RNAseq data and proteins from the genome of *Megachile rotundata*. L_RNA_Scaffolder –v 1.0.0 [10] and PEP_Scaffolder –v 1.0.0 [11] were used for this purpose with default parameters. The N50 of the resulting –v3 assembly increased to 604 kb (S14 Table)

**Re-mapping read pairs against –v3 assembly**

The trimmed paired-end and mate-pair reads of each library were mapped separately in paired mode against the final genome assembly using BWA with all default parameters. Only RF oriented reads from mate-pair libraries were used for re-mapping (S15 Table). The mappings were sorted by position with samtools sort –v 1.1.0 [12] and mapping statistics were obtained using QualiMap bamqc –v 2.2.0 [13]. Accumulation of insert sizes on the lower end of the distribution and higher mean coverage than expected can be explained by mate pairs that cannot span repetitive regions.

**Assembly validation**

The final –v3 assembly was validated by adopting core gene content analysis using the Core Eukaryotic Gene Mapping Algorithm, CEGMA –v 2.5.0 [14] and the Benchmarking Universal Single-Copy Orthologs, BUSCO –v 3.0.0 [15] approaches (S16 Table and S4 Table respectively).

**Repeat library**

A repeat library was created using RepeatModeler –v 1.0.4 [16]. RepeatModeler collapsed all repeats into 685 repeat families. Most of these families are classified as ‘unknown’ with 62, 42 and 29 belonging to the DNA, LTR and LINE repeat families respectively (S5 Fig). K-mer distribution of short reads indicated 42 MB of repetitive sequence in the *O. bicornis* genome. Short-reads were mapped back to the repeat library (as for genome re-mapping above), with a total length of 973,108 bp, obtained from RepeatModeler. The alignment was performed using the BWA mem –v 0.7.12-r1039 command and default parameters. In total, 153,741,711 reads were mapped back to the repeat library covering 59.9% of the short read dataset (38435427750 bp).

**Annotation**

The first round of gene prediction was performed using BRAKER –v 2.1.0 [17]. RNA-seq reads (see below) were mapped against the masked –v3 assembly to determine exon-intron junctions using TopHat –v 2.1.1 [18] with default settings with the resulting BAM file fed to BRAKER. The BRAKER pipeline resulted in 36,287 gene models. In a second round of annotation, three iterations of the MAKER2 2.31.8 [19] pipeline, combined with MPICH2 (<http://www.mpich.org/>), were performed. Initially, an AUGUSTUS species model was computed and trained using the –v3 assembly. *O. bicornis* gene features in GFF format from BUSCO were used as input. BUSCO was run on the –v3 assembly using the Arthropoda dataset together with the option --long but with other parameters unchanged. From a CEGMA run on the –v3 assembly a SNAP –v 2006-07-28 [20] model was built using the script cegma2zff, from MAKER2 distribution, and the SNAP scripts fathom (fathom genome.ann genome.dna -categorize 1000 && fathom -export 1000 -plus uni.ann uni.dna), forge (export.ann export.dna) and hmm-assembler.pl. In addition, a Genemark –v 4.32 [21] model was built from self-training (--ES) on the –v3 assembly. The –v3 assembly, the AUGUSTUS species model, transcripts from RNA-seq data (see below), the custom repeat library and the HMM models from SNAP and GeneMark were used as input for the first MAKER iteration. The options est2genome and protein2genome were switched off. Furthermore, the minimum protein length reported was set to 10 amino acids. After the first iteration the GFF file for the whole assembly was extracted using MAKER gff3_merge, converted with maker2zff and a new HMM model built for SNAP as above. The AUGUSTUS species model was retrained locally by first converting with the SNAP script zff2gff3.pl (zff2gff3.pl genome.ann | perl -plne 's/\t(\S+)$/\t\.\t$1/') and second the autoAug.pl script from AUGUSTUS –v 3.2.2 [22]. The input for the autoAug.pl was the –v3 assembly, the trained AUGUSTUS species model and the gff3 file created from the first MAKER iteration. For the second MAKER iteration the SNAP HMM model from CEGMA was exchanged to that created from the output of the first iteration and the updated AUGUSTUS species model used as input. The minimum protein length was raised to 30 amino acids. After retraining as above a third MAKER iteration was performed. Following this, a third round of annotation was performed using PASA –v 2.3.3 [23]. Transcripts from the sex-specific RNA-seq dataset, the -v3 genome assembly and the gff3 file obtained from the BRAKER run were used as input in PASA with default parameters. All the gff3 files obtained from BRAKER, MAKER2 and PASA were then used collectively as input to EVM –v 1.1.1 [24] resulting in 15,065 high quality gene models. Annotation comparison was also performed as a final step to update the existing EVM gene models. The EVM produced annotation file, in GFF format, was loaded in the PASA database using the script Load_Current_Gene_Annotations.dbi. GFF3 data adapters were included in the PASA distribution. A comparison was performed between PASA alignment assemblies and the pre-existing PASA database gene annotations, to identify cases where updates can be automatically performed to gene structures in order to incorporate the transcript alignments. This comparison resulted in a new GFF3 file containing the PASA-updated version of the genome annotation, including those gene models successfully updated by PASA, and those that remained untouched. A diagrammatic representation of the annotation workflow is available in S6 Fig.

The final GFF3 resulted in 14858 genes coding 18479 proteins with lengths between 30 AA and 18337 AA. Gene lengths are between 150 and 393458 bp and sum to a total of 77239236 bp which corresponds to 36.3% of the total assembly size and 41.75% of haploid genome size of 185 MB. These genes contain 42588032 bp of exons with an average of 6 exons per gene. Exon lengths range from 1 bp to 18571 bp and sum to a total of 42588032 which corresponds to 13.2% of the total assembly size (S3 Table). Functional annotation of all protein sets with Gene Ontology (GO) terms was performed with BLAST2GO [25] and InterProScan 5 [26], using default parameters.

**Ortholog analysis**

Protein sets of 6 bee species (*Apis florea*, *Apis mellifera*, *Bombus impatiens*, *Bombus terrestris*, *Megachile rotundata* and *Osmia bicornis*) were used to perform ortholog inference using OrthoFinder –v 0.7.1 [27] with default parameters (S17 Table and S18 Table).

***De novo* transcriptome assembly**

*De novo* assembly of RNAseq data derived from male and female bees (see main methods) was carried out using the Trinity Software Suite –v 2.0.6 [28] (S19 Table). The transcriptome assembly was used for whole genome annotation as detailed above.

**Supplementary references**

1. Andrews S (2010) FastQC: a quality control tool for high throughput sequence data. Available online at: <http://www.bioinformatics.babraham.ac.uk/projects/fastqc>
2. O’Connell, Jared, et al. (2015) NxTrim: optimized trimming of Illumina mate pair reads. *Bioinformatics* 31:2035-2037.
3. Li H (2013) Aligning sequence reads, clone sequences and assembly contigs with BWA-MEM. *arXiv*:1303.3997v2.
4. Wysoker A, Tibbetts K, Fennell T (2013) Picard tools version 1.90 <http://picard.sourceforge.net>
5. Andrews S (2011) FastQ Screen. Available online at: <http://www.bioinformatics.babraham.ac.uk/projects/fastq_screen/>
6. Schmieder R, Edwards R (2011) Fast identification and removal of sequence contamination from genomic and metagenomic datasets. *PLoS ONE* 6:e17288.
7. Vurture GW, Sedlazeck FJ, Nattestad M, Underwood CJ, Fang H, Gurtowski J, Schatz MC (2017) GenomeScope: fast reference-free genome profiling from short reads. *Bioinformatics* 33:2202-2204.
8. DISCOVAR: Assemble genomes, find variants. [https://www.broadinstitute.org/software/discovar/blog. Accessed 3 October 2015](https://www.broadinstitute.org/software/discovar/blog.%20Accessed%203%20October%202015).
9. Pryszcz LP, Gabaldón T (2016) Redundans: an assembly pipeline for highly heterozygous genomes. *Nucleic Acids Res* 44(12):e113.
10. Xue W, Li JT, Zhu YP, Hou GY, Kong XF, Kuang YY, Sun XW (2013) L_RNA_scaffolder: scaffolding genomes with transcripts. *BMC Genomics* 14:604.
11. Zhu BH, Song YN, Xue W, Xu GC, Xiao J, Sun MY, Sun XW, Li JT (2016) PEP_scaffolder: using (homologous) proteins to scaffold genomes. *Bioinformatics* 32:3193-3195.
12. Li H, et al. (2009) The sequence alignment/map format and SAMtools. *Bioinformatics* 25:2078–2079.
13. Okonechnikov K, Conesa A, García-Alcalde F (2016) Qualimap 2: advanced multi-sample quality control for high-throughput sequencing data. *Bioinformatics* 32:292-294.
14. Parra G, Bradnam K, Korf I (2007) CEGMA: a pipeline to accurately annotate core genes in eukaryotic genomes. *Bioinformatics* 23:1061-1067.
15. Simão FA, Waterhouse RM, Ioannidis P, Kriventseva EV, Zdobnov EM (2015) BUSCO: assessing genome assembly and annotation completeness with single-copy orthologs. *Bioinformatics* 31:3210-3212.
16. Smit A, Hubley R (2012) RepeatModeler - 1.0.5. Institute for Systems Biology, <http://www.repeatmasker.org/RepeatModeler.html>
17. Hoff KJ, Lange S, Lomsadze A, Borodovsky M, Stanke M (2016) BRAKER1: Unsupervised RNA-Seq-based genome annotation with GeneMark-ET and AUGUSTUS. *Bioinformatics* 32:767-769.
18. Trapnell C, Pachter L, Salzberg SL (2009) TopHat: discovering splice junctions with RNA-Seq. *Bioinformatics* 25:1105-1111.
19. Holt C, Yandell M (2011) MAKER2: an annotation pipeline and genome-database management tool for second-generation genome projects. *BMC Bioinformatics* 12:491.
20. Korf I (2004) Gene finding in novel Genomes. *BMC Bioinformatics* 5:59.
21. Lomsadze A, Ter-Hovhannisyan V, Chernoff YO, Borodovsky M (2005) Gene identification in novel eukaryotic genomes by self-training algorithm. *Nucleic Acids* *Res* 33:6494-6506.
22. Stanke M, Schöffmann O, Morgenstern B, Waack S. Waack (2006) Gene prediction in eukaryotes with a generalized hidden Markov model that uses hints from external sources. *BMC Bioinformatics* 7:62.
23. Haas BJ et al. (2003) Improving the Arabidopsis genome annotation using maximal transcript alignment assemblies. *Nucleic Acids Res* 31:5654-5666.
24. Haas et al. (2008) Automated eukaryotic gene structure annotation using EVidenceModeler and the Program to Assemble Spliced Alignments. *Genome Biol* 9:R7.
25. Conesa A et al. (2005) Blast2GO: a universal tool for annotation, visualization and analysis in functional genomics research. *Bioinformatics* 21:3674–3676.
26. Jones P et al. (2014) InterProScan 5: genome-scale protein function classification. *Bioinformatics* 30:1236-1240.
27. Emms DM, Kelly S (2015) OrthoFinder: solving fundamental biases in whole genome comparisons dramatically improves orthogroup inference accuracy. *Genome Biol* 16:157.
28. Haas BJ et al. (2013) De novo transcript sequence reconstruction from RNA-seq using the Trinity platform for reference generation and analysis. *Nat Protoc* 8:1494-1512.
